# Roles for Structural Biology in the Discovery of Drugs and Agrochemicals Targeting Sterol 14α-Demethylases

**DOI:** 10.3390/jof7020067

**Published:** 2021-01-20

**Authors:** Brian C. Monk, Mikhail V. Keniya

**Affiliations:** Department of Oral Sciences, Sir John Walsh Research Institute, University of Otago, Dunedin 9016, New Zealand; mikhail.keniya@otago.ac.nz

**Keywords:** antifungal drug discovery, CYP51, sterol 14α-demethylase, azole drugs, azole agrochemicals, structure-directed drug design, in silico screens, pharmacophore, molecular docking

## Abstract

Antifungal drugs and antifungal agrochemicals have significant limitations. These include several unintended consequences of their use including the growing importance of intrinsic and acquired resistance. These problems underpin an increasingly urgent need to improve the existing classes of antifungals and to discover novel antifungals. Structural insights into drug targets and their complexes with both substrates and inhibitory ligands increase opportunity for the discovery of more effective antifungals. Implementation of this promise, which requires multiple skill sets, is beginning to yield candidates from discovery programs that could more quickly find their place in the clinic. This review will describe how structural biology is providing information for the improvement and discovery of inhibitors targeting the essential fungal enzyme sterol 14α-demethylase.

## 1. Introduction

### 1.1. Changing Paradigms in Antifungal Discovery

The remarkable genetic and phenotypic diversity of fungal pathogens, the multifaceted relationships and genetic similarities these eukaryotes share with their human or agricultural hosts, as well as the economic costs of antifungal development, make the urgently needed expansion of the current armamentarium of antifungal drugs and fungicidal agrochemicals a daunting challenge [1,2,3]. Since the 1950s, the use of quantitative structure activity relationship (QSAR) and phenotypic screens have yielded a modest number of antifungal classes, with a subset of these substances finding practical application in the clinic or field [4]. The most important members of the latter group include the polyenes, azoles, echinocandins, allylamines and pyrimidine analogs used against human pathogens while the azoles, succinate dehydrogenase inhibitors, anilinopyrimidines, coenzyme Q inhibitors, morpholines and methyl-benzimidazole carbamates have been used widely as pesticides directed against plant pathogens [2]. Without these agents fungal infections would have elicited devastating impacts on human health and food resources. There would have been more than the 1.5 million deaths attributed per annum to fungal disease and millions more would have died due to the critical role of agrochemical fungicides in maintaining food security [5]. However, few antifungals have broad-spectrum application, either because of innate resistance displayed by some fungal species or due to the rapid development of acquired resistance on antifungal exposure. A particularly significant concern is that exposure to azole agrochemicals has led to the selection of strains of the major pathogen *Aspergillus fumigatus* that are resistant to azole drugs used in medicine [6]. In addition, some antifungals have undesirable side effects due to either drug-drug interactions or because their unavoidable impact on the host or ecosystems cannot be mitigated with careful monitoring and husbandry.

Technical innovations such as molecular genetic tools and expression systems that apply information gleaned in an ongoing era of genomic exploration, together with insight into fungal phylogeny and physiology, can now assist antifungal discovery and development. While not a panacea, the availability to the scientific community via the Protein Data Bank (PDB) of pertinent high resolution structures of drug targets and their host homologues, including the conformations adopted by inhibitory ligands in complex with their targets, plus off-target structural insights, complement and focus drug discovery paradigms. Such paradigms include the use of phenotypic screens with genetically engineered target constructs as a prelude to studies with more complex clinical or field isolates of disease causing fungi. The use of libraries of target-ligand structures can facilitate the computer-based improvement of existing antifungals, including those compounds whose activity is affected by target-based resistance determinants [7]. Such structures can also be used to identify pharmacophores which are defined as ensembles of steric and electronic features that ensure optimal supramolecular interactions with a specific biologic target and cause a biological response [8]. As described by Dinic et al., pharmacophore modeling can be of three types; (i) ligand-based, where the pharmacophore hypothesis is built from a collection of active (or possibly inactive) ligands without using target information; (ii) target-based, where the pharmacophore hypothesis uses structural data from the receptor only; and (iii) complex-based, where the pharmacophore model uses structural data of protein-ligand complexes [9]. Of these three approaches, the most powerful uses pharmacophores based on drug targets in complex with substrates and/or inhibitory ligands. Pharmacophore identification enables rapid screening in silico of compound libraries for physiochemically-related ligands with significant potential to be active in experimental tests. Computer-based docking with target structures of libraries of compounds, drug-like fragments, or even compounds identified via pharmacophore-based screens, can be used to obtain quantitative indications of the most likely inhibitory ligands or identify possible components of such ligands [10]. Exploration in silico of broad expanses of chemical space with pharmacophores and/or molecular docking techniques offers opportunity to efficiently accelerate the discovery of novel antifungal candidates directed against structurally resolved targets or surrogate models. The accompanying papers and reviews in this issue will address the present and potential impact of structural biology on antifungal discovery. This review will primarily provide an overview of structural biology related to the azole drugs and agrochemicals. We are fortunate that the PDB now contains a large number of high-resolution crystal structures of fungal and host CYP51s in complex with known antifungals, antifungal candidates and, in some cases, with enzyme substrates.

Downstream of drug discovery, the application in the clinic or field of novel classes of antifungals is likely to remain a complex and long-term aspiration for most academics and biotechnology companies. This is because the skills and economic resources of the pharmaceutical or agrochemical industries are needed to address the multiple requirements of drug development. These include meeting ADME-Tox requirements, completing pharmacokinetic studies, and complying with stringent regulatory challenges before drug and agrochemical candidates can reach the market. Meanwhile, improved prevention strategies, faster diagnostics that identify fungi at the species level and highlight drug susceptibilities/resistances, together with the case-by-case application of existing antifungal medications and agrochemicals, are being used to minimize the incidence of serious fungal disease and slow the seemingly inevitable emergence of drug resistance. Of particular importance is the judicious use of prophylaxis, prevention and treatment strategies that minimize opportunity for the development and expansion of drug resistance, and to plan for and be able to apply practical alternative therapies should resistance occur.

### 1.2. The Fungal Kingdom in the Anthropocene

The fungal kingdom is estimated to comprise ~2.2–3.8 million species [11] and apart from a small proportion of exceptions fungi play key and usually unseen roles in the well-being of the planet. Of the ~120,000 fungal species identified thus far, a few hundred have a proven negative impact on human health, while fewer than 8000 species constitute known threats to food security, or ecosystems including soils, grasslands, forests, rivers, lakes and seas [1]. It is widely understood that human activity in the Anthropocene has created the opportunity for particular fungal species to operate as pathogens in naive ecosystems or on individual hosts due to comorbidities or medical interventions that remove physical or immunological barriers to infection. These opportunists include fungal species capable of innate or acquired resistance, or that show significant tolerance of the synthetic and semisynthetic chemicals used to kill fungi or that block their growth sufficiently to render them susceptible to host immune surveillance. Less well understood are the unintended consequences of the use of antifungals, e.g., the impact of antifungal pesticide residues on human well-being, on our food chain, and on non-pathogenic fungi that contribute to the vitality of ecosystems [2].

### 1.3. Commensals and Opportunist Fungal Pathogens of Humans

Physical barriers such as the skin and actively protective epithelial and mucosal surfaces limit normal colonization of humans to a relatively small group of significant fungal pathogens [5]. These include dermatophytes such as the *Trichophyton* species that can cause ringworm and athlete’s foot, and the commensals *Candida albicans* and *Candida glabrata* that usually live harmlessly on the skin, in the mouth and the gastrointestinal, respiratory and reproductive tracts. Oral infections are, however, relatively common, with blockage of host salivary flow (e.g., in Sjogren’s disease and due to dry mouth induced by drug treatments or head and neck irradiation) or abrasion of the oral epithelial lining by ill-fitting dentures in the elderly [12]. Such problems provide opportunity for oral thrush or denture stomatitis caused by *C. albicans*. Antibiotic treatments that eliminate bacterial infections can destroy much of the protective endogenous microbiota in the mouth and digestive tract and lead to opportunistic fungal overgrowth. The role of oestrogens and hormonal changes in the menstrual cycle and immune modulation in pregnancy increases the susceptibility of females to mucosal infections of the reproductive tract. Disease- or medically-induced immune deficiency or chemotherapy affecting the lining of the gastrointestinal tract of leukemics can drastically weaken protective responses on mucosal surfaces. The resultant overgrowth of commensal fungal species can result in oral or vaginal thrush or invasive fungal disease among leukemics. Considered by many to be an opportunist, *C. glabrata* is less pathogenic than *C. albicans* but is about 10 times more likely to be resistant to one or more antifungal classes and causes systemic infections that are more likely to be lethal. This, in part, is due to instability of its compact haploid genome and susceptibility to damage of a key DNA repair system [13,14]. In recent decades, non-albicans *Candida* species, especially *C. glabrata* and *Candida parapsilosis*, have replaced *C. albicans* as dominant causes of candidemia and invasive candidasis in some regions of the globe [15]. Most other pathogenic fungi that cause opportunistic infections in humans are soil saprophytes. These organisms are usually cleared by immune competent individuals and are not normally transmitted from person to person. In contrast, the emergence of *C. parapsilosis* is probably due to its transmission via contact between patients and health professionals or caregivers. *Candida auris* has recently emerged as a global threat that poses considerable risk due to its drug resistant phenotypes, including resistance to multiple antifungal drug classes (e.g., azoles, echinocandins and polyene antibiotics), and persistence in hospital and aged care settings [16,17,18]. In recent years, there have been about a million cases per annum of cryptococcosis caused by *Cryptococcus neoformans*. This disease has been associated primarily with AIDS patients in Sub-Saharan Africa and, despite the use of relatively inexpensive drugs, its requirement for a complex long-term (1 year) antifungal treatment regime often gives poor outcomes, with about 100,000 affected individuals dying each year [19,20]. *Cryptococcus gattii*, while originally associated with the tropics (Central America), has caused outbreaks in the West Coast of the United States and Canada among otherwise healthy individuals [21]. There are about 20,000 cases of Valley fever (coccidiodomycosis) reported annually in the United States but the numbers affected may be substantially larger [22]. This disease is caused predominantly by spores of the environmental pathogen *Coccidioides immitis* which enter the lungs. While healthy individuals in the United States, Central and South America can contract Valley fever, the infection mostly affects immune compromised individuals. *Aspergillus fumigatus* is the mold most frequently responsible for fungal disease. Infections by *A. fumigatus* occur widely in the community. These include asthma and tuberculosis related respiratory diseases (such as allergic pulmonary aspergillosis, severe asthma with fungal sensitisation and chronic pulmonary aspergillosis) that collectively affect about 20 million individuals [5,23]. Life threatening invasive *A. fumigatus* infections have high mortality rates and are complicated, not only by the innate resistance of *A. fumigatus* to fluconazole (FLC), but also because of acquired resistance, including cross-resistance, to the drug of choice voriconazole (VCZ). The cross-resistance problem seems to have emerged at multiple locations across the globe due to exposure to azole agrochemicals [24]. In addition, the use of corticosteroid and anticancer therapies is associated with about 200,000 patients developing invasive aspergillosis each year [25]. Serious fungal infections of humans caused by the mucormycetes are particularly common in third world agricultural settings and frequently lead to disfiguring tissue damage, blindness and ultimately lethal meningitis [26]. These infections are becoming increasing common because type II diabetics are readily infected and are particularly concerning because the mucormycetes are intrinsically resistant to FLC and VCZ. Visual impairment is an all too frequent outcome of eye infections in Asia and Africa caused by filamentous fungi, with *Aspergillus* and *Fusarium* spp. affecting about 10% of such patients. Finally, the treatment of lung infections with immunosuppressive therapies significantly increases the risk of sequelae involving invasive fungi infections. For example, treatment of severe COVID-19 disease with immunosuppressive therapies such as dexamethasone and IL6 inhibitors has been reported to be associated with a 26.7% incidence of invasive fungal infections, with pulmonary aspergillosis accounting for 14% of these infections [27]. Early treatment with antifungal drugs, directed by strategic mycological testing, appears to be protective in this context [28].

Superficial infections caused by *C. albicans* and the dermatophytes are usually readily treated with standard antifungal drugs such as terbinafine, nystatin, the imidazoles and over-the-counter FLC preparations. Life-threatening invasive fungal infections associated with AIDS or modern medical techniques that subvert natural defences are an important concern as they require the more potent and more recently developed azoles such as VCZ, posaconazole (PCZ) and isavuconazole (IVC) or echinocandins such as caspofungin and micafungin. While the echinocandins are much more expensive than the azole drugs, they have become the first line treatments of yeast infections in Western medicine. However, some non-*Aspergillus* molds, *Cryptococcus* spp. and the vast majority of *Fusarium* and *Mucorales* spp. are not susceptible to these agents [29]. PCZ, which is arguably the most broad-spectrum of the azole drugs, usually has good activity against yeast, molds and muco-mycetes, while *Fusarium* spp. are problematic. PCZ has often been the azole drug of choice for salvage therapy, but its inhibition of liver detoxifying enzymes can restrict its use. As rates of drug metabolism and liver cytochrome P450 susceptibilities vary between individuals, therapeutic drug monitoring is important when administering PCZ, ITC, VCZ or terbinafine [30,31]. PCZ use needs to be monitored closely and it should not be used in some patients due to drug-drug interactions. Patients treated with terbinafine, VCZ and itraconazole (ITC) similarly need close monitoring to avoid toxic side-effects. ITC and PCZ inhibit CYP3A4 activity and VCZ is metabolized by CYP3A4 and CYP2C19. Terbinafine is metabolized by about seven liver CYP450 enzymes, and although this has little or no effect on the metabolism of many characteristic CYP450 substrates, it is, however, a competitive inhibitor of the CYP2D6m [32]. It has been suggested that IVC, which is delivered as the prodrug isavuconazolium, does not require therapeutic drug monitoring. Exceptions may include patients in therapeutic failure or unexplained or moderate hepatotoxicity, as well as those who are noncompliant, obese, or receiving concomitant medications predicted to reduce IVC concentrations, or are aged <18 years [33].

### 1.4. Fungal Disease in the Environment

The emergence of destructive fungal infections of forest trees [34] and of amphibians [35] are driven by multiple factors that may be generally applicable to the etiology of fungal pathogenesis and, in some cases, to the acquisition of antifungal resistance. International trade that moves hosts or fungi into naive environments, as well as pollution and climate change associated with human activity that affect the fitness of host or fungi, have been linked with increased susceptibility and the spread of fungal diseases. Such diseases include Dutch elm disease (caused by *Ophiostoma* sp.), Chestnut blight (*Cryphonectria parasitica*), Ash dieback (*Hymenoscyphus fraxineus*), Kauri dieback (convergent evolution of fungus-like *Phytophthora agathidicida)*, Myrtle rust (*Austropuccinia psidii*), and the widespread decline of over 500 amphibian species due to chytridiomycosis caused by *Batrachochytrium dendrobatidis* and in one case by *Batrachochytrium salamandrivorans*. Additional factors that may increase susceptibility to fungal infection include hybridization between fungal species [36], new associations with disease vectors, widespread monoculture of clonal hosts such as rice, soybean and banana, and antifungal resistance [1,2,3].

In contrast to our disease-oriented understanding of why commensal fungi such as *Candida* spp. become pathogens of humans, many fungi take part in mutually beneficial relationships critical for normal plant growth and the colonization of ecosystems, e.g., mycorrhizae and endophytes [2]. Disruption of such relationships through the incursion of non-native fungi or of resistant phytopathogens that are then controlled by using large quantities of more potent or persistent antifungals should be viewed with some trepidation, particularly in Europe where fungicides are heavily applied and their impact on the biota of soils and the aquatic systems needs more study [37]. Similar concerns may apply to the human mycobiome, a system about which we have limited functional knowledge. For example, the human gut mycobiome normally has low diversity compared to the bacterial component of these microbiomes. The fungal component of the gut microbiome is dominated by the yeast genera *Saccharomyces*, *Malassezia*, and *Candida* [38]. This population appears to be readily modified by dietary or environmental fungi [39], with the vaginal and oral mycobiomes acting as inoculants [40,41], and by bacterial species present in the gut [42]. Although antifungal prophylaxis is recommended for neutro-penics undergoing chemotherapy [43], the indirect effects of antifungal agents on the gut microbiome or antibacterial agents on the gut mycobiome are poorly understood. It is of interest that efficient mating in *C. albicans* (reviewed by Correia et al. [44]) occurs by a two-step process that can occur in the gastrointestinal tract. This involves the conversion to a homozygous mating type cell followed by a transition to the opaque state. After mating, a return to a diploid state requires concerted chromosome loss, providing an important source of genetic variability for this opportunistic pathogen that may play a role in the development of antifungal resistance.

### 1.5. Fungal Disease and Modern Agriculture

Susceptibility to fungal disease is a major problem for modern agriculture, with fungicides used to improve crop yield, quality and shelf life [45]. Primary crops such as rice, wheat, soybean, maize, sugarcane, potatoes, grapes, bananas, coffee and pip fruit are all susceptible to specific fungal diseases. These often require complex husbandry including multiple interventions with a variety of pesticides that are often applied as mixtures to ensure efficacy [2]. Limited genetic diversity in crop monocultures increases the likelihood that food security will be threatened by epidemics of phytopathogens, especially those resistant to antifungal pesticides [1]. This threat is most pressing for major crops such as rice, wheat, and soybean, especially in temperate zones where there are high fungicide requirements. It is estimated that almost one half of the land in Europe used for crops and viticulture is treated annually with azole fungicides. If use of the azole class was to cease in Europe due to fungicide resistance or concerns about their effects on the human endocrine system [46], Europe’s agricultural self-sufficiency and competitiveness in the global wheat market may be compromised. For example, fungicides are needed to sustain cereal cropping in Ireland and possibly other Northern European countries (reviewed in [47]). Some other fungal threats to global food security include organisms affecting stored crops such as peanuts, potatoes, apples and tropical fruits. These routinely receive pre-harvest treatment with azole agrochemicals.

Until humanity can arrest and reverse the current acceleration of environmental change or obtain acceptable genetically modified crops resistant to fungal pathogens, fungal disease will remain a major and increasingly difficult challenge that has to be fought on multiple fronts, including the judicious use of agrochemicals such as the azole fungicides.

## 2. Discovery of Antifungal Drugs and Agrochemicals

### 2.1. Some Practical Considerations for Drug Discovery

The economics of drug development is a major impediment that has restricted interest in obtaining new classes of antifungals. The pharmaceutical and agrochemical industries naturally prefer broad-spectrum antifungals that are readily and inexpensively manufactured. In contrast, narrow spectrum antifungals require sufficiently large markets to meet the costs of their development. Olorofim, the recently discovered orotide antifungal that affects molds and thermally dimorphic species but not yeast, will be an important exception if it can circumvent this limitation [48,49]. Drug-related side effects should be minimal, both in host organisms and in the environment. Satisfying this requirement needs extensive and expensive clinical or field trials. A more recent realization is that the use of agrochemical pesticides can compromise the use of medicinal antifungals [6,24]. This is especially problematic for the existing azole pesticides that appear to have driven the selection worldwide of *A. fumigatus* strains resistant to azoles used in the clinic. Overcoming this problem may require the development and application of distinctly different classes of antifungals for these separate markets. Finally, where possible antifungals need to be designed to circumvent mechanisms, such as the induction of drug tolerance, that ultimately enable the stable genetic changes characteristic of acquired drug resistance [50].

### 2.2. Antifungal Drugs Used in the Clinic and Agriculture

The ability to identify effective broad spectrum antimicrobials specific for fungi has been limited because fungi are eukaryotes like their human and plant hosts and hence share many key metabolic enzymes that have maintained high levels of similarity during evolution from a common ancestor over the last billion years. Some chinks in this armour have been exploited. Medicines have been developed that inactivate enzymes specific to fungi (e.g., glucan synthase, the target of the echinocandins), permeabilize membranes by binding to a fungal specific metabolic product (e.g., ergosterol, the target of the polyenes) or take advantage of amino acid substitutions that confer a clinically useful level of specific binding to a fungal homologue of an enzyme found in the host (e.g., CYP51 or sterol 14α-demethylase, the target of the azoles; squalene monooxygenase, the target of the allylamines). Obtaining broad-spectrum antifungals is likely to remain problematic because of the intrinsic resistance of some fungal groups to specific classes or subclasses of antifungal agent. For example, Olorofim inhibits dihydroorotate dehydrogenase in the de novo pyrimidine biosynthesis pathway of molds, but not yeast [48]. In contrast, FLC is effective against yeast but the molds and mucormycetes are innately resistant, while the closely structurally related azole VCZ is very effective against yeast and is the drug of choice for infections caused by the mold *A. fumigatus*, but mucormycetes are innately resistant [29]. The mechanisms responsible for these forms of innate resistance are being resolved and are expected to inform structure-based drug discovery [51,52]. PCZ arguably remains the most broad-spectrum antifungal of the azoles currently used in the clinic. It inhibits the growth of yeast, molds and mucormycetes but drug–drug interactions can limit its use.

**Figure 1:**
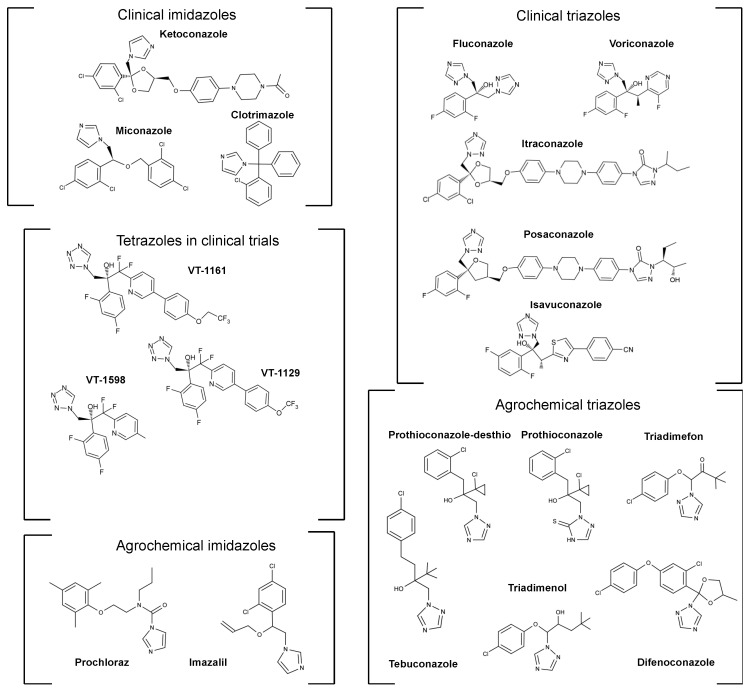
Representative azole drugs, agro-chemicals and compounds in clinical development.

A six decade history of the application of QSAR technology and phenotypic screens has led to the current generations of marketed azole agrochemicals and drugs as well as some novel azole drugs in clinical trials (see Figure 1 for representative compounds) [53]. The azole drugs target the cytochrome P450 enzyme referred to as sterol 14α-demethylase (CYP51) or lanosterol 14α-demethylase (LDM, Erg11) and inhibit the early step in the biosynthesis of the fungal-specific sterol ergosterol which it catalyzes. This point was not fully proven until 1987 when it was found that the activity of *Saccharomyces cerevisiae* LDM was competitively but completely inhibited by ketoconazole at a concentration equal to that of the enzyme [54]. An important contemporaneous finding was that mammalian cells are much less sensitive than fungal cells to azole drugs such as ITC [55].

The roles of the fungal-specific sterol ergosterol and the regulation of its biosynthesis were reviewed recently [56]. Ergosterol is the main sterol found in fungal membranes. It is involved in the maintenance of membrane structural integrity, fluidity and permeability, and the activity of membrane bound enzymes. It is required for cell proliferation, may have a role in maintenance of mitochondrial DNA and is important for stress adaption including to the effects of temperature, low sugar, alcohol and oxidative stress. In animals the primary sterol is instead cholesterol, which acts as a precursor for vitamin D, bile acids and steroid hormones, while in plants the phytosterols are required for growth and differentiation and are involved in stress adaption. Clearly, inhibitors of fungal CYP51 should be specific and not inactivate host CYP51s or other cytochrome P450 enzymes, although the human diet can provide sufficient sterols even when hydroxy-methyl-glutamate-CoA reductase is inhibited due to the therapeutic use of statins.

The first azole drugs, Clotrimazole and miconazole, were licensed for use as topical agents against dermatophytes and *Candida* spp. [57,58]. Because it was effective against dermatophytes and yeast, plus dimorphic and filamentous fungi in vitro, clotrimazole was considered as a possible broad-spectrum replacement for amphotericin B [59]. The therapy was unsuccessful due to significant side effects and important pharmacokinetic issues [60]. Clotrimazole remains a useful topical agent. Developed also as an intravenously administered drug, Miconazole has a similar spectrum of action as clotrimazole but is ineffective against molds [61]. Like clotrimazole, it had side effects, including cardiac arrhythmia when administered in large doses [62]. The toxicity of clotrimazole and miconazole is thought to be due to their potent inhibition of the liver enzymes CYP2C9, CYP2C19 and CYP3A4 [63,64]. Miconazole is now restricted to topical applications.

First marketed by Janssen Pharmaceutica in 1981, oral formulations of the imidazole Ketoconazole (KTC) were better tolerated and proved more successful than their predecessors [57]. In addition to poor solubility, disadvantages include associated hepatotoxicity, endocrine dysregulation and unpredictable drug interactions [65,66,67]. KTC also inhibits CYP3A4 [68], an enzyme responsible for the metabolism of ~37% of clinically used drugs, followed by CYP2C9 (17%), CYP2D6 (15%), CYP2C19 (10%) CYP1A2 (9%) and CYP2C8 (6%) [69]. In addition, liver metabolism of KTC produces toxic cyanide adducts that give significant side effects [70]. The use of KTC to treat systemic fungal infections was superseded with the introduction of the triazoles in the early 1980s. It is thought that the triazole heterocycle has advantages over the imidazole ring because it is more metabolically stable and binds more weakly to host cytochrome P450s [71]. FLC and ITC were the first triazole drugs in medical use [53]. FLC is water-soluble, is bioavailable and used in both oral and intravenous formulations. It is effective against *Candida* spp. and *C. neoformans* and is better tolerated than KTC. Its penetration of the blood brain barrier enables treatment of cryptococcal meningitis, but molds such as *A. fumigatus* have innate resistance to FLC. VCZ, which was discovered at Pfizer by synthesizing over 1200 azole analogs, substantially overcame this problem to become the drug of choice for treating invasive aspergillosis [72]. ITC and its subsequently developed congener PCZ are fungicidal for *A. fumigatus* and, although used in prophylaxis for some patients, both antifungals are poorly soluble in water and have low CNS penetration.

The use of FLC as a prophylactic measure and in treating systemic fungal infections has been limited by the increased incidence of infections caused by fungal pathogens less susceptible to FLC and the emergence of intrinsically resistant species. FLC monotherapy of AIDS patients, who are highly susceptible to *Candida* or *Cryptococcus* infections, precipitated a rapid increase in the incidence of azole resistant clinical isolates of *C. albicans* until Highly Active Antiretroviral Therapy (HAART) was introduced in the 1990s [73]. FLC can still be used as a first-line treatment and prophylaxis against invasive candidiasis, with VCZ and ITC providing optional treatments. The use of PCZ is restricted to therapy of oropharyngeal and oesophageal candidiasis, while unpredictability in bioavailability and trough plasma concentrations means it is only applied in the prophylaxis of some high-risk patients. Although FLC remains a cornerstone treatment for cryptococcal disease, especially in resource poor regions where flucytosine is too expensive, the incidence of acquired FLC resistance among patients with relapse in this disease is increasing [74]. An increasing incidence of infections caused by innately VCZ resistant mucormycetes, especially among diabetics, plus acquisition of PCZ resistance, are major concerns due to the facial damage, blindness and death caused by these pathogens [26].

IVC is azole drug most recently approved for systemic fungal infections. In Phase 3 clinical trials IVC and VCZ were comparably effective in treating invasive mold disease [58]. In vitro IVC works well against *Candida* spp., including FLC resistant strains and *Aspergillus* spp., but is less effective against less common molds such as *Fusarium* and *Scedosporium* spp. [75,76]. IVC is administered as the water-soluble prodrug isavuconazonium sulfate (Cresemba), either orally or intravenously, to patients with invasive aspergillosis or mucor-mycosis. The prodrug is cleaved by plasma esterases into IVC and an inactive by-product. IVC has more favorable pharmacokinetic properties and fewer side effects than other triazoles.

Viamet Pharmaceuticals has developed several tetrazole antifungals, most notably VT-1161, VT-1129 and VT-1598. One of the four nitrogen atoms in the tetrazole heterocycle coordinates to the heme iron of CYP51s in a significantly weaker interaction than comparable interactions involving the imidazole or triazole heterocycles [77,78]. Drug interactions for VT-1161 with liver CYP450s are predicted to be low due to its >2000-fold selectivity towards the *C. albicans* CYP51 (CaCYP51) compared to human CYP51 (HsCYP51), [79]. VT-1161 has completed Stage II clinical trials. VT-1161 is well tolerated by individuals with mild fungal infections, effectively treats recurrent vulvovaginal candidiasis and fungal nail infections [80], but its effect on patients with weak immune systems is not known. VT-1129, which has a tail shorter by one carbon atom than its congener VT-1161 (Figure 1), has activity against *C. neoformans* and *C. gattii* [81] and binds preferentially to fungal CYP51 compared to HsCYP51 [82]. Despite an absence of animal studies, the drug was fast tracked into clinical trials, with a view to treating cryptococcal meningitis. VT-1598 is also in clinical trials. It has a similar head group, but its tail is slightly longer and chemically different to the other two tetrazoles. VT-1598 is active in murine models of infection by *Coccidioides posadasii* and *C. immitis* [83] and in vitro studies have shown it to be active against *Candida*, *Cryptococcus* and *Aspergillus* spp. [84].

The semi-synthetic echinocandin drugs (e.g., caspofungin and micafungin) are widely used in the Western world for the prophylaxis and treatment of *Candida* and *Aspergillus* infections despite being expensive, not orally bioavailable. Due to poor neurological penetration, they are not effective in treating some disseminated infections, e.g., cryptococcal meningitis [85]. The repertoire of triazole antifungals still provides drug of choice treatments for many serious fungal infections. This is because they are relatively inexpensive, usually have modest and readily monitored side effects, are bioavailable, and can used for prophylaxis or some longer term treatments [86]. The low cost and accessibility of azole drugs such as FLC is very important in resource poor areas where HIV is endemic and cryptococcal meningitis kills a high proportion (<60%) of affected individuals [19].

### 2.3. Intrinsic and Acquired Resistance to Azole Drugs

The acquisition of azole resistance among intrinsically susceptible fungi is a significant problem, especially in patients who receive extensive or repeated antifungal prophylaxis or treatments. The general mechanisms that can lead to azole resistant clinical isolates of *C. albicans* are relatively well understood, especially following seminal research with the azole resistant daughter progeny of azole susceptible strains recovered from individual patients [73]. These and subsequent studies have provided important exemplars for other *Candida* species and for molds.

Like other sterols, such as cholesterol found in humans, the incorporation of the fungal-specific sterol ergosterol into the plasma membrane is thought to modulate its thickness, fluidity, permeability and microdomain formation [87]. Preferential localization of ergosterol (detected using filipin) in large sterol rich domains found at tips of mating projections, hyphae and at septation sites, and in association with sphingolipids during the formation of the smaller lipid raft domains, confers membrane heterogeneity that is expected to affect the distribution and activity of a range of key plasma membrane associated proteins [88]. More recently the yeast plasma membrane has been described as containing numerous domains [89]. These include the large membrane compartment of the plasma membrane proton pump Pma1 in which proteins diffuse rapidly and both endocytosis and cell wall biosynthesis occur, and about 50 small punctate patches of the membrane compartment of the arginine permease Can1. A further set of important punctate patches is contributed by the membrane compartment of the TORC2 kinase which is involved in cell polarity and sphingolipid biosynthesis. Inhibition of lanosterol 14α-demethylase causes a rapid decline in the levels of ergosterol, the terminal product of the fungal sterol biosynthetic pathways [90]. This leads to a fungistatic increase in membrane permeability, negative effects on the activity of some membrane associated enzymes and the production of toxic fecosterols [91]. The development of azole resistance in *C. albicans* to widely used drugs such as FLC, VCZ and PCZ appears to be a multistep process that produces levels of drug resistance that can ultimately result in MIC values 100-fold greater than the susceptibility of naive wild type strains [73]. The process can be viewed as commencing with the development of drug tolerance [50]. This can involve azole drugs binding to and activating transcriptional regulators such as Upc2 and Tac1 that significantly increase the expression of the genes encoding LDM (*Erg11*, *CYP51*) and both ABC (e.g., CaCdr1 and CaCdr2) and MFS (e.g., CaMdr1) drug efflux pumps [92]. In some instances, aneuploidy may occur in the chromosome containing the *ERG11* and *TAC1*, resulting in elevated expression of CaCYP51 and drug efflux pumps [93,94]. Further exposure to azole drugs can select for mutations in *ERG11* that produce a target enzyme with reduced susceptibility to all azole drugs or, in some cases, to a restricted group of the azole drugs (discussed in a subsequent section). *C. albicans* is diploid and has two *ERG11* alleles and these appear to be highly susceptible to mutation. Numerous non-synonymous SNPs have been detected in Ca*ERG11*, but only a limited number of single mutations or specific combinations of these mutations have been confirmed as conferring azole resistance due to modification of azole binding affinity by CaCYP51 [95,96,97,98]. Some of these mutations, such as CaCYP51 Y132F, are commonly mimicked in other fungal pathogens including *C. parasilosis* and *C. tropicalis* [99,100]. Mutations equivalent to Y132F in some fungal species may also need to be supplemented with mutations that increase enzyme stability and/or modification of the CYP51 promoter to increase expression of the mutant enzyme e.g., *A. fumigatus* CYP51A TR_46_/Y121F/T289A [24]. Given sufficient time, gain-of-function mutations in transcriptional regulators enable the constitutive overexpression of both CYP51 and the drug efflux pumps. *C. krusei* is naturally resistant to azole drugs and appears to achieve this by having three *ERG11* genes and inducing key drug efflux pumps. In some cases, a loss of function of the *ERG3*, which prevents the alternative metabolism of lanosterol into formation of toxic fecosterols, allows *C. albicans* to continue to grow in the presence of azole drugs [91].

The molds and mucormycetes have two genes (*CYP51A* and *CYP51B*, *CYP51 F1* and *CYP51 F5*, respectively) that encode sterol 14α-demethylases with differential susceptibilities to azole drugs. There is now good evidence to indicate that *CYP51A* in the mold *A. fumigatus* confers intrinsic resistance to FLC [52] and *CYP51 F5* in the mucormycete *Rhizopus arrhizus* confers intrinsic resistance to both FLC and VCZ [51]. The molecular basis of these phenotypes is discussed in subsequent sections.

### 2.4. Azoles Used in Agriculture

The first azole antifungals used as agrochemicals (denoted as sterol demethylase inhibitors or DMIs) were introduced in the 1970s. Unlike the medical azoles, the imidazoles and triazoles were released at about the same time. The imidazoles imazilil and prochloraz and the triazoles triadimefon and triadimenol were among the first azole fungicides used in agriculture [101]. Economically important fungal diseases of plants treated by azoles include wheat rusts caused by *Puccinia* spp., septoria leaf blotch in wheat caused by *Z. tritici* (also known as *Mycosphaerella graminicola*), rice blast disease caused by *Magnaporthe oryzae*, powdery mildew of grasses caused by *Blumeria graminis*, black sigatoka in bananas caused by *Mycosphaerella musicola*, Panama disease or fusarium wilt in bananas caused by *Fusarium oxysporum* and the mycotoxin producing fungal species such as *Aspergillus flavus* and *Aspergillus parasiticus*. The DMIs account for a large proportion of fungicide use because they are cost-effective and broad spectrum [102]. The ongoing evolution of the DMIs has also been particularly important in the light of the even more rapid appearance of high level resistance, usually within a few seasons, to most other classes of fungicides that have been introduced, including the succinate dehydrogenase inhibitors (SDIs), anilinopyrimidines, Coenzyme Q inhibitors (QoIs), morpholines and methyl-benzimidazole carbamates. Despite target-based resistance to individual DMIs also occurring, their judicious use in mixtures and their structural diversity has provided opportunity to subvert existing resistance genotypes. As described below, the use of different azoles has been associated with changes in azole susceptibility and the spectrum of mutations associated with *Z. tritici* CYP51 (ZtCYP51, reviewed in [103]). The triazoles tebuconazole, epoxiconazole and the more recently introduced (2000) prothioconazole have been among the most widely used azoles in the UK, Netherlands and Denmark [104]. The prodrug Prothioconazole is converted to an active desthio form that inhibits CYP51s, as has been shown for both *C. albicans* and *S. cerevisiae* enzymes [105,106]. Prolonged use of DMIs can result in tolerance and acquired resistance often involving mutations in CYP51s [102,103]. By 2008, seventeen different amino acid substitutions, often in combinations, had been found in ZtCYP51, including mimicry of modifications that confer azole resistance in *C. albicans* CYP51 [107].

Long term and/or excessive use of the DMI agrochemicals have been associated with resistance among fungal phytopathogens, i.e., the acquisition of non-synonymous mutations in *CYP51* genes, overexpression of CYP51s, multiple (including innately resistant) CYP51 paralogs or increased efflux of the compounds from fungal cells via ATP binding cassette (ABC) and Major Facilitator Superfamily (MFS) drug transporters. A limited representation of phytopathogenic fungi that have acquired reduced susceptibility to azoles include strains of *Zymoseptoria tritici* in wheat, *Blumeria graminis* in barley and wheat, *Phakopsora pachyrhizi* in soybean, *Mycosphaerella fijiensis* in banana, and *Botrytis cinerea* in fruits and vegetables [108]. In this review, we focus in brief on two major examples: *Z. tritici* and *P. pachyrhizi*.

*Z. tritici*, a cause of leaf blotch in winter wheat in Europe and also reported in Australia, compromises growth of the plants [109]. Homology modeling of ZtCYP51 using the structure of *S. cerevisiae* LDM as a template (PDB 4LXJ), suggested that the enzyme is substrate specific, binding eburicol as a preferred substrate. As the binding of the lanosterol in the template structure used for the modeling study now appears to be incorrect, this interpretation of the binding of eburicol should be reevaluated [110]. ZtCyp51 was shown to have a temperature dependent catalytic efficiency in presence of its native NADPH cytochrome P450 reductase enzyme (CPR) [111]. Some mutations found in ZtCYP51 correspond to the acquired mutations found in CaCYP51. The ZtCYP51 Y137F mutant was dominant in Europe in the 1990s, possibly due to the extensive use from 1970 of the small secondary alcohol containing triazole triadimenol. This mutation has also been found in powdery mildews of grapes and cereals, wheat brown rust and in the black sigatoka fungus [103]. More recent mutations detected in ZtCYP51, such as I381V, V136A and S524T, confer resistance to tebuconazole, prochloraz, and prothioconazole, respectively [112]. Mutations in the fungus specific loop such as G464S also confer azole resistance or are required in conjunction with other mutations to confer resistance [103]. Strains bearing such mutations appear to have evolved independently worldwide in response to the more recently developed azoles, while ZtCYP51 Y137F mutants are now rare [103]. This suggests that selective use of different classes of antifungals could minimize the development azole resistance conferred by ZtCYP51.

*P. pachyrhizi* is a cause of Asian soybean rust. Since its detection in Japan in 1902, a virulent form of *P. pachyrhizi* has spread globally to China, countries in South East Asia, and Australia, Africa and the Americas. *P. pachyrhizi* has been a major risk to the South American soybean crop since its detection there in 2001 [113]. A crop loss worth about US$2 billion for Brazil was reported in 2003 [114]. Some isolates of *P. pachyrhizi* from Brazil have been found to be resistant to DMIs. Overexpression or amino acid substitutions such as Y131F, Y131H, K142R, F120L, I145F and I475T in *P. pachyrhizi* CYP51 (PzCYP51) appear to confer the resistance. An homology model of PzCYP51, generated from the crystal structure of the catalytic domain of HsCYP51 (PDB ID: 3LD6) as template and docked with azoles such as cyproconazole, epoxiconazole, metconazole and tebuconazole, was used to elucidate the possible effects of these mutations [115]. Several PzCYP51 mutations structurally align with CYP51 mutations in clinical isolates and in agricultural isolates of *U. necator*, *B. graminis*, *P. triticana* and *M. fijiensis* [116].

## 3. Structural Biology and Functional Analysis of Lanosterol 14α-Demethylase

### 3.1. Obtaining CYP51 Crystal Structures

Determining crystal structures of membrane proteins has several challenges. Obtaining sufficient amounts of enzyme for purification and crystallization often requires heterologous overexpression of an engineered recombinant protein in a suitable host such as *Escherichia coli* or *S. cerevisiae.* Solubilizing functional membrane proteins requires appropriate physiochemical conditions together with a detergent that enables retention of bioactivity during purification and is compatible with crystallization [117]. In some instances, a more favorable detergent or lipid surrogate such as lipid micelles or nano-disks may be exchanged in a subsequent step. Purification from detergent extracts to a sufficient level of purity for crystallography (ideally mono-dispersity) usually requires multiple chromatographic steps, most often involving at least both affinity chromatography and size exclusion chromatography.

**Figure 2:**
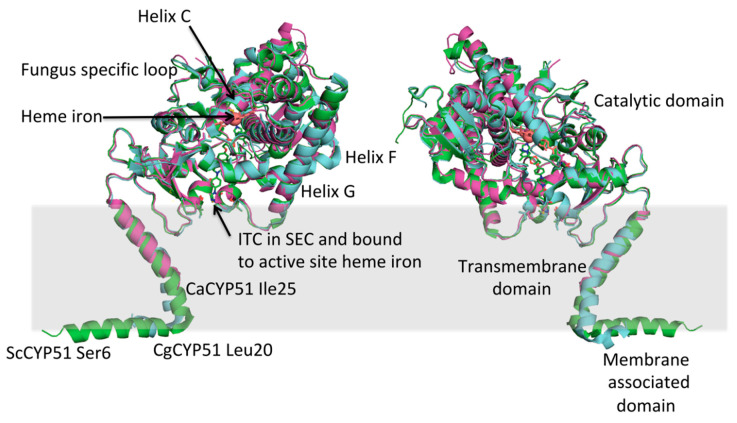
Overlaid crystal structures of *S. cerevisiae*, *C. glabrata* and *C. albicans* lanosterol 14α-demethylases in complex with itraconazole (ITC). The crystal structures of these fungal CYP51 enzymes, viewed from opposites sides, have the same fold and are likely to share the same orientation with respect to the endoplasmic reticulum (shown in grey). The ScCYP51 structure (visualized from Ser6) is in green, CgCYP51 (visualized from Leu20) in light blue and CaCYP51 (visualized from Ile25) in purple. While the entire primary sequence of the fungus specific loop is can be modeled in ScCYP51, significant portions of the more N-terminal part of this surface structure could not be modeled in the *C. glabrata* and *C. albicans* CYP51s. The ITC triazole group interacts identically with the heme iron (red sphere) and very similarly with the ligand-binding pocket in all three structures but with slight differences in orientation of this ligand at the mouth of the substrate entry channel (SEC).

The first fungal CYP51 to be crystallized and structurally resolved (Figure 2) was obtained using recombinant full-length *S. cerevisiae* CYP51 (ScCYP51) with a C-terminal hexa-histidine tag that was constitutively overexpressed in yeast, extracted from crude membrane preparations using the detergent decyl-maltoside, and purified using Ni-NTA affinity chromatography and size exclusion chromatography [118]. The Protein Data Bank now contains over 30 high-resolution crystal structures for full-length fungal CYP51s. These include ScCYP51 in complex with lanosterol, the pseudo-substrate estriol, the clinical azoles ITC [118], FLC [119], VCZ, PCZ [120] and VT-1161 [121], the agro-chemicals difenconazole, fluquinconazole, prochloraz, prothioconazole-desthio and tebuconazole [106], as well as structures obtained for several key mutations introduced into the yeast enzyme [122]. More recently crystal structures were obtained for full-length *C. albicans* and *C. glabrata* CYP51 (CgCYP51) in complex with ITC (Figure 2) [123,124]. Also available are truncated structures: CaCYP51 catalytic domain in complex with VT -1161 and PCZ [77] and *A. fumigatus* CYP51B (AfCYP51B) catalytic domain in complex with VNI [125] and VT-1598 [126]. Despite sequence differences amongst fungal CYP51s, the catalytic domain in all published X-ray crystal structures retain the characteristic cytochrome P450 fold and most have a relatively rigid active site and substrate entry channel (SEC) within the ligand-binding pocket (LBP).

The crystals structures obtained with recombinant full-length *S. cerevisiae*, *C. glabrata* and *C. albicans* CYP51, together with chemical labeling and computer studies, show the enzyme to be a bitopic membrane protein with a transmembrane helix that spans endoplasmic reticulum once (Figure 2). Partially visualized in the *S. cerevisiae* and *C. glabrata* structures but not the *C. albicans* structure, a short membrane-associated amphipathic sequence of variable length at the N-terminus of the enzyme is located at endoplasmic reticulum luminal surface. For all three structures, a catalytic domain of over 450 amino acids in the cytosol is associated with one face of the C-terminal third of the transmembrane helix. The catalytic domain is oriented with respect to the lipid bilayer via hydrophobic and specific hydrogen bond interactions with the transmembrane helix and contacts with the lipid bilayer [118].

### 3.2. Mechanistic Features of CYP51 Enzymes

Like all cytochrome P450 enzymes, the CYP51s are monooxygenases with a catalytic domain that has a highly conserved and unique fold [127]. Fungal LDMs and their eburicol metabolizing isoforms are members of the CYP51 family, the most phylogenetically ancient of the CYP450 families [128]. The cytochrome P450 enzymes have evolved to carry out specific metabolic reactions that help produce and modify metabolites including sterols, some lipids and vitamins, or they can act as more broadly specific activators or detoxifiers of drugs. The eukaryotic CYP51 family has maintained its primary function of metabolizing sterol precursors at an early step in the sterol biosynthetic pathway, with specificity for a limited range of substrates that includes lanosterol, eburicol and obtusifoliol. The sterol 14α-demethylase catalytic domain has a buried iron containing porphyrin (heme) that forms one surface within the deeply embedded catalytic site. The enzyme NADPH-cytochrome P450 reductase donates electrons to this active site heme by docking with the enzyme surface in the neighborhood of helix C, the C-terminal end of a large external loop (denoted the fungal specific loop in fungal systems) and in proximity of the heme bulge containing the conserved cysteine that forms a fifth ligand of the heme iron. The bound substrate undergoes three reaction cycles each involving the formation of the highly reactive Fe^IV^-peroxo group, resulting in the removal of the 14α-methyl group and the insertion of a C14-C15 double bond in the substrate, the production of formic acid, the consumption of protons and the loss of water molecules [129].

The ligand-binding pocket (LBP) includes the catalytic site and the substrate entry channel (SEC) that reaches to the cytosolic surface of the endoplasmic reticulum near the membrane spanning helix and in close proximity of two beta sheets and the F” helix in the loop between the F and G helices. The LBP also includes a putative product exit channel (PPEC) that diverges from the SEC. The PPEC appears to present a site at the LDM surface adjacent to the membrane for the product of LDM activity to interact with the series of enzymes downstream in the ergosterol biosynthetic pathway [118]. These enzymes include the Erg24 reductase and the Erg25-Erg27 C4-demethylase system mounted on the scaffold Erg28 protein. Several studies suggest that *S. cerevisiae* is part of an even larger Ergosome complex that also includes the Erg6 C24-methyl transferase required late in the ergosterol biosynthetic pathway in yeast and the acyl-CoA:sterol acyltransferase Are1 required for sterol esterification and viability [130,131,132,133]. In molds, a cognate Erg6 converts lanosterol to the sterol 14α-demethylase substrate eburicol while the plant-specific substrate otusifoliol is formed from the triterpenoid precursor cycloartenol by the actions of C24-methyl transferase, C4-demethylase and a cyclopropyl-isomerase.

A gated pathway (S channel) in *S. cerevisiae* LDM (reversed in the CYP51s in comparison with other classes of cytochrome P450s) is thought to use residue D233 in helix F, and H317 in helix I, for the unidirectional uptake of substrate protons from near the membrane and into the active site beside helix I [125,134]. In crystal structures with inhibitory ligands these two residues make a salt bridge. A water (potential hydronium ion accepted from H317) is positioned to hydrogen bond with the main chain carbonyls of M313 and G314 and the main chain amides of H317 and T318 [118]. Located at the slight kink in helix I, this water may have a role in proton delivery to the LBP via the S channel gated by H317 and D233 [77]. The complex with water is found for ScCYP51 in a low occupancy precatalytic complex with lanosterol (PDB ID: 4XLJ) but is absent in yeast CYP51s in complex with azole drugs that coordinate with the heme (e.g., with ITC in PDB ID: 5EQB) despite retention of the helix I kink. The absence of the water appears to be due to the proximity of the di-halogenated phenyl head group and the triazole to helix I when azole drugs like FLC, VCZ, ITC or PCZ are bound to the heme iron. It is not known how oxygen accesses the active site but the interaction of bimolecular oxygen with the heme iron can be visualized in the *S. cerevisiae* LDM precatalytic complex with lanosterol. The LBP includes, in addition to the SEC, the PPEC [118]. The PPEC has an open conformation in the *C. glabrata* LDM in complex with ITC (PDB ID: 5JLC) and in the *C. albicans* LDM catalytic domain in complex with ITC or PCZ (PDB IDs: 5V5Z, 5FSA) but not in the *C. albicans* LDM catalytic domain in complex with VT-1161 (PDB ID: 5TZ1). This conformational difference in the *C. albicans* structures involves movement of residues around the PPEC, especially F233. The *S. cerevisiae* LDM PPEC usually has an open conformation, but this can be closed off due to the movement of residues beside the PPEC, most notably by F241 (structurally aligned with *C. albicans* LDM F233). In addition, the conformation of the conserved *S. cerevisiae* LDM M509 residue seems to affect the boundary between the active site and the SEC. In a mutant apo structure (PDB IDs 5ESI) or in complex with VCZ (PDB ID 5HS1), but not with other azole drugs, the M509 side chain closes off the SEC instead of just lining it.

Membrane bound cytochrome P450s, which locate to the endoplasmic reticulum or mitochondria of eukaryotic cells, have catalytic domains with a comparable fold but were classified as significantly structurally different from the soluble P450s that occur in bacteria [135]. This finding was based primarily on the interaction of the heme ring C propionate with the helices A-B loop in the case of the membrane bound enzymes and with helix C for the soluble enzymes. The membrane bound CYP51 enzymes provide an illustrative exception to this generalization. Both soluble and membrane bound enzymes in this ancient cytochrome P450 family have their heme ring C propionate in an ionic interaction with a basic residue in helix C (K143 in ScCYP51). A second factor that discriminates between soluble and membrane-bound cytochrome P450s is the increased length and more complex disposition of the F-G helix region in the membrane bound cytochrome P450s.

### 3.3. Ligand Binding by CYP51 Enzymes

A feature invoked for rational antifungal design is the similarity across phyla of CYP51 structures and the absence of major structural rearrangements in complexes with various inhibitory ligands or structural analogs [7,134]. However, structures obtained for full-length and truncated CYP51s in complex with the short-tailed tetrazole inhibitor VT-1161 and the long-tailed triazole inhibitor PCZ suggest that the disposition of the mouth of the substrate entry channel required for broad spectrum antifungal activity may be compromised in truncated structures liganded with this short-tailed azole due to structure distorting inter-subunit crystal lattice interactions [121]. The use of full-length LDM crystal structures as templates may therefore be an important consideration for the in silico discovery of azole drugs.

**Figure 3:**
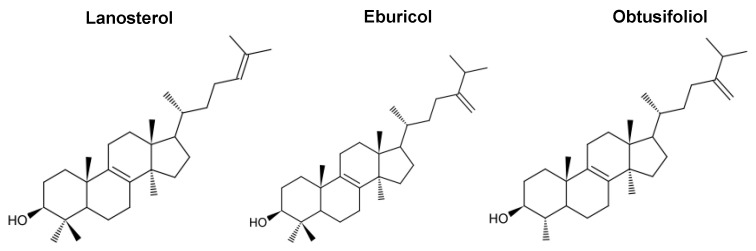
The structures of CYP51 substrates.

Poor substrate binding with both truncated and full-length CYP51 molecules have led to conflicting proposals for substrate orientation. The likely orientation of sterol substrates (Figure 3) was recently clarified using an I105F mutant of *Trypanosoma cruzi* CYP51 (TzCYP51) [110]. The mutation converted a fungi-like eburicol-specific CYP51 to a plant-like obtusifoliol-specific enzyme but with substrate occupancy increased to ~85%. This allowed reliable visualization of this substrate in the binding cavity formed by the B-C loop, helix C and helix I, with the obtusifoliol hydroxyl group oriented into the substrate access channel. Comparable visualization of the substrate lanosterol was achieved with the human CYP51 D231A H314A mutant that has the salt bridge involved in proton delivery oblated [136]. Furthermore, with productive substrate binding by both the protozoan and human enzyme, a significant reorientation of helix C occurred. In particular the heme propionate-helix C ionic linkage via a lysine residue was lost and the freed basic side chain projected outwards from the enzyme surface.

The use of docking techniques and molecular dynamics has modeled possible interactions between membrane bound mammalian NADPH-cytochrome P450 reductase (CPR) and membrane bound liver enzyme CYP1A1 [137]. The mimicking of complementary ionic, van der Waals and hydrophobic interactions between the CPR FMN domain and the residues on the B, C and L-helices, the J-K loop and the loop structure near the CYP1A1 heme, plus the inclusion of a hydrogen bond between the FMN phosphate group and the Q139 sidechain in helix C, appeared to enable efficient electron transfer to the heme. Crystallographic and NMR analysis of the bacterial cytochrome P450s, the camphor binding CYP101A and mycinacin biosynthetic enzyme MycG, indicate the movement of particular secondary structure elements during substrate binding [138,139]. This finding has been validated by site-directed mutagenesis experiments and used to suggest a generally conserved mechanism for substrate binding and recognition in the Cytochrome P450 superfamily.

In CYP51s, accommodation of the substrate in a catalytically competent position is now expected to drive reorientation of helix C and CPR binding, close the substrate entrance, and activate of the proton relay machinery via F-F″-G arm repositioning and the His-acid salt-bridge opening required for the O–O bond heterolysis that produces compound I. This process has been suggested to prepare the CYP51 catalytic machinery for the three consecutive reaction cycles characteristic of this class of cytochrome P450. It occurs without the substrate release after its first and second monooxygenation reactions, distinguishing it from most other cytochrome P450s [136].

The crystal structures of ScCYP51 suggest a channel located between the heme ring D propionate and the protein surface that may facilitate the removal of product water molecules into the cytosol [140]. This channel has been modeled to contain five hydrogen-bonded waters in ScCYP51 in complex with lanosterol and four hydrogen-bonded waters when ITC is the ligand. A hydrogen bond between a water molecule and the propionate is found in both structures, while both structures retained most but not all hydrogen bonding contacts between the waters and the protein. The hydrogen bond networks include contributions from the main chain carbonyls and amides of G465, the main chain carbonyls of V112, F113, A115, L117, V120 and A122, the side chain guanidine of R385 and the main chain nitrogen and side chain imidazole of H468. No comparable channel was seen from the heme ring C propionate. In contrast, the HsCYP51 structure in a catalytically competent complex with lanosterol suggests a similar water channel from heme ring D plus an additional water channel involving at least four water molecules that extends from the heme ring C propionate to the enzyme surface. The latter channel involves hydrogen bonding with the main chain and imidazole side chain of H447, the amide side chain of N149, the main chain amide of N121 and the carboxyl side chain of E122 [110]. Residues H447 in HsCYP51 and H468 in ScCYP51 structurally align and their differing contributions in the drug and substrate bound structures suggest the heme bulge and its interaction with the cognate NADPH-cytochrome P450 reductase may play an important role in the conformation of the channels needed for product water removal. Finally, how the formate produced in the CYP51 active is released has not been established. Elucidating the mechanistic features of water and formate loss will require further insight into enzyme conformation.

### 3.4. The CYP51 Ligand-Binding Pocket

Crystal structures obtained for full-length LDMs from *S. cerevisiae*, *C. glabrata* and *C. albicans*, in complex with a variety of azole-containing antifungal compounds, indicate that the LBP has about 46 amino acids contributing to its surface (Table 1). Only four residues contributing to the surface of the ScCYP51 active site are conserved in all fungal CYP51s that we have analyzed and are retained in both human and plant hosts. These residues are Y126 and F134 in helix B, Q150 in helix C and H317 in helix I of ScCYP51. Their locations within the active site are consistent with involvement in sterol 14α-demethylase catalytic function such as provision of hydronium ions (H317), controlling the conformation of the B helix—BC loop—C helix region required for substrate binding, and regulating substrate entry and product egress. Site-directed mutagenesis experiments are needed to test such ideas.

Comparison of the human CYP51 D231A H314A structure in complex with lanosterol and ScCYP51 in complex with azole drugs suggests that some additional amino acid residues near the active site might contribute to the LBP on lanosterol binding by the yeast enzyme. These would include A122 in helix B′, L212 in the turn between helix D and E, plus L312, S319 and T322 in helix I. Whether these changes due to the reaction cycle can be exploited in antifungal discovery is an open question. For example, none of the homologous residues in human CYP51 crystal structure (L309, S316 and T320) are within 4 Å of lanosterol and give a small and relatively inaccessible extension of the LBP between helix I and the heme.

In the case of ScCYP51, 24 of the LBP amino acid residues are within 4 Å of the long-tailed triazole ITC (PDB 5EQB). Although the ScCYP51 crystal structures show 12 LBP residues are within 4 Å of FLC (PDB 4WMZ) and 10 within the same distance of VCZ (PDB 5HS1), this difference is predominantly due to FLC lying significantly (0.5 Å) closer than VCZ to helix I and to Y140 in the BC-loop. It is therefore likely that FLC will be more susceptible to the conformational changes during the reaction cycle and associated features that determine substrate specificity.

Although it can be shown that the triazole drugs in clinical use inhibit more strongly fungal CYP51s than their mammalian CYP51 counterpart or key liver drug metabolizing cytochrome P450s, the structural basis of these differences has yet to be fully elucidated. After identifying the region in HsCYP51 immediately prior to the conserved H314 in helix I as conformationally flexible, Friggeri et al. used a T318I mutation to stiffen this helix [141]. The T318I mutation in HsCYP51 was reported to increase the amount of main chain hydrogen bonding in helix I and made the purified enzyme more susceptible to the azole inhibitor VNI and longer-tailed VNI derivatives. It was rationalized that the human enzyme is naturally more resistant to azole drugs because it is more conformationally dynamic and, together with a shorter F-G loop affecting substrate entry, has a higher catalytic rate and hence a greater capability for substrates to compete with azole drugs that rely on binding within the active site.

**Figure 4:**
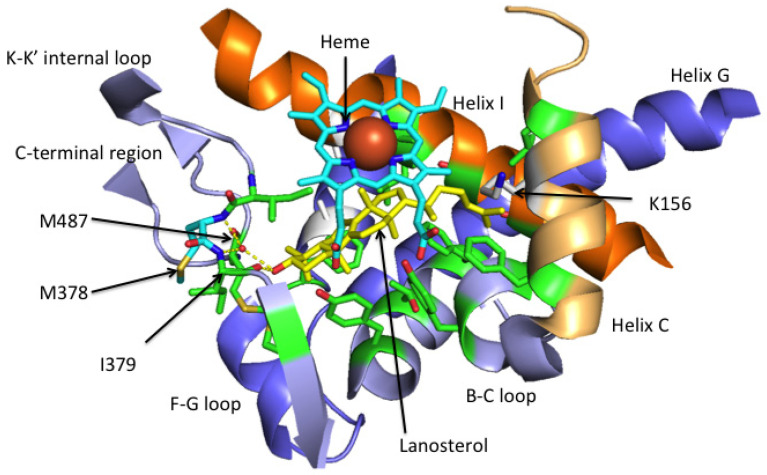
Lanosterol in the ligand-binding pocket (LBP) of CYP51. Relevant portions of the human HsCYP51 crystal structure (Protein Data Bank (PDB) 6UEZ) are shown. Residues within 4 Å of lanosterol (yellow) are shown with carbon atoms in green. These 16 residues are found in helix I, helix B, helix C, the B-C loop, the K-K′ internal loop and the C-terminal region. Proton channel S mutations, D231A in helix F and H314A in nearby helix I, are shown in white. Upon lanosterol binding helix C has changed conformation slightly, the K156 side chain loses its ionic interaction with the heme propionate C and becomes exposed into the enzyme surface. The K-K′ internal loop I379 main chain carbonyl hydrogen bonds with the OH of lanosterol. The main chain amides of M378 and I379 plus the main chain carbonyl of M487 in the C-terminal region form a water-mediated hydrogen bond network with the hydroxyl of lanosterol. The 14α-methyl group of lanosterol lies in proximity of the heme iron (large red ball) in a catalytically competent position.

**Figure 5:**
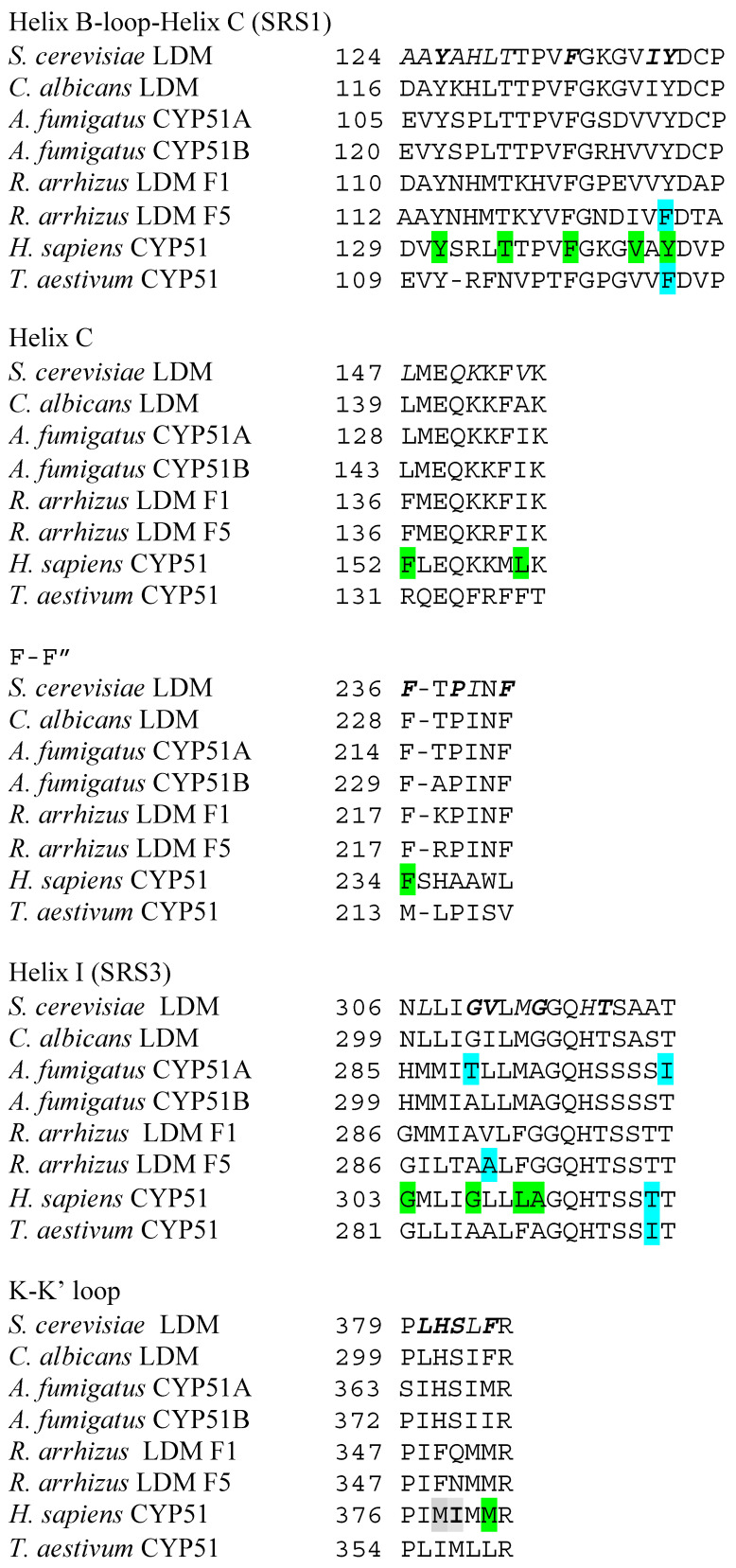

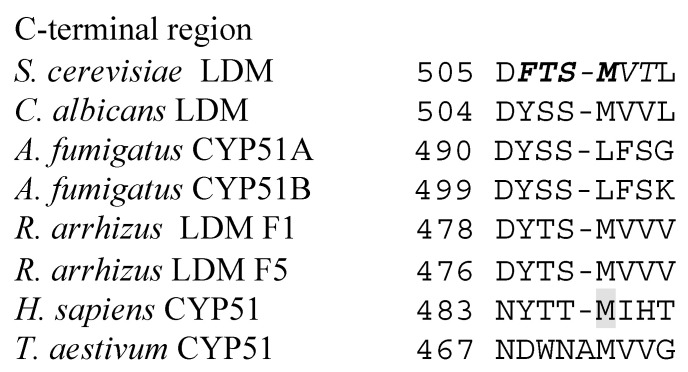
Comparison of itraconazole (ITC) and lanosterol binding plus residues that confer innate resistance to azole drugs in eukaryotic CYP51s. Residues within ScCYP51 LBP are highlighted in italics and those within 4 Å of ITC are highlighted in bold. Residues within 4 Å of lanosterol in the HsCYP51 D231A H314A mutant catalytic domain that has high substrate occupancy are highlighted in green. Residues with their main chain amide (M378 and I379) or main chain carbonyl (M487) involved in a water-mediated hydrogen bond network with the 3-hydroxyl of lanosterol are highlighted in gray while the residue (I379) using its main chain carbonyl to make a direct hydrogen bond with lanosterol is highlighted in gray and in bold. Residues where alignments and experimental work indicate possible roles in substrate specificity and/or causes of innate resistance to azole drugs are highlighted in light blue. Selected residues identified as relevant to innate azole resistance representative of molds are illustrated using *A. fumigatus* and for mucormycetes they are illustrated using *R. arrhizus*.

CYP51 enzymes have specificity for a limited number of sterols and this aspect has yet to be fully elucidated. Lanosterol is the natural substrate of yeast and mammalian (including human) CYP51s but they can also bind eburicol. Molds can use both lanosterol and eburicol as substrates. For example, expression studies in *S. cerevisiae* with its native CYP51 deleted show that the CYP51A enzyme of *A. fumigatus* requires eburicol while its CYP51B enzyme is able to use lanosterol. Plant CYP51s use otusifoliol as their natural substrate. The structure of HsCYP51 in complex with lanosterol and primary sequence comparisons within the CYP51 LBP offer some useful clues about substrate specificity (Figure 4 and Figure 5). The alignment of the tail of lanosterol in its complex with HsCYP51 beside the N-terminal half of helix I suggests that LBP residues in this region could affect substrate specificity. It is therefore reasonable to hypothesize that T289 in the N-terminal half of helix I of AfCYP51A, which aligns with A303 in AfCYP51B, G310 in ScCYP51, and G307 in HsCYP51 may explain the preference of AfCYP51A for eburicol as substrate while AfCYP51B, yeast and mammalian CYP51s prefer lanosterol. The equivalent in the plant representative *T. aestevium* is A285 and, although the hydrophobic tails of eburicol and otusifoliol contain an identically disposed double bond, the I helix in plants and *A. fumigatus* contain downstream residues that may make the I helix stiffer than their AfCYP51B, yeast or human counterparts, thereby affecting substrate affinity. In addition, the plant (*T. aestevium*) CYP51 binding cavity contains F125 instead of the Y in the B-C loop. In other species the Y residue forms a hydrogen bond with the heme ring C propionate which, in turn, has an ionic interaction with the residue equivalent to K156 in the human enzyme. In addition, the *T. aestevium* CYP51 binding cavity includes R131 instead of an F in helix C that could favorably interact with the obtusifoliol tail double bond, and M213 instead of F in the short F-F″ loop that could more favorably fill the space vacated by the absence of one of the two 4-methyl groups.

It was established previously that AfCYP51A but not CYP51B is responsible for the innate resistance of *A. fumigatus* to FLC [142]. Despite being outside the LBP, except during substrate binding, it also is likely that the I301 substitution in CYP51A (T318 in CYP51B and an aligned T residue in the CYP51s of other non-molds) contributes to the reduced susceptibility to FLC [52]. Contrasting results were obtained when Sagatova (PhD thesis, University of Otago) made the equivalent T322I mutation in ScCYP51. Crystal structures showed the mutational loss of the side chain hydroxyl led to the loss of hydrogen bonds with a water molecule and the carbonyl of the main chain of T318 but the conformation of helix I was unchanged and FLC could still be bound as ligand. In addition, the susceptibilities to FLC, VCZ or ITC of the strain expressing ScCYP51 T322I were actually unchanged compared with those obtained for the wild type strain. The only significant difference observed in Type II binding studies with affinity purified recombinant enzymes was that the ΔA_max_ values for binding of FLC, VCZ, ITC and PCZ by the mutant enzymes were 2/3 to 3/4 those of the wild type enzyme, possibly indicative of reduced inhibitor occupancy. Collectively, these results suggest that an additional feature found in AfCYP51A, and not found in its CYP51B counterpart or in ScCYP51, may contribute to the innate FLC resistance conferred by AfCYP51A. For example, the bulky and polar T289 side chain of AfCYP51A (aligns with A302 in AfCYP51B) and the difluorophenyl head group of FLC are within 3 Å and this could thereby confer the selective resistance to FLC. As noted above, FLC closely interacts with helix I while the other triazole drugs, including VCZ, have more extensive interactions with other parts of the LBP. Thus, AfCYP51A T289 and I301 may both contribute to the innate FLC resistance of molds. It is interesting to note that the innately pan-azole resistant *Scedosporium*-like species *Lomentospora prolificans* was recently found to have a CYP51 sequence containing residues that align with azole resistance mutations G138S, M220I and T289A in AfCYP51A [143]. The G138S mutation is outside the LBP in helix C, M220I lies at the mouth of the SEC and could confer pan-azole resistance, while TR_46_/T289A/Y121F strains have acquired VCZ resistance.

The mucormycete *Rhizopus arrhizus* is intrinsically resistant to FLC and VCZ and expresses two isoforms of CYP51, F1 and F5. Structural modeling suggests the F5 isoform confers innate resistance to both FLC and VCZ due the combination of the Y129F substitution in the BC loop and a V291A substitution in helix I compared with the F1 isoform [51]. Biochemical experiments with wild type and mutant CYP51 F5 isoforms co-expressed with their cognate reductase in yeast membranes indicate that susceptibility to FLC and VCZ but not PCZ is due to the B-C loop Y129F substitution, which leads to a loss of water-mediated hydrogen bond interactions between the hydroxyl groups of FLC or VCZ and the protein, plus the helix I V291A substitution.

## 4. Antifungal Discovery and Design

### 4.1. Can Better Antifungals Be Designed?

The range of Protein Data Bank crystal structures of fungal CYP51s in complex with azole drugs and agrochemicals map how these compounds bind within the LBP. This detailed insight into interactions with both the heme and individual amino acid residues in the LBP of wild type and mutant enzymes is helping the design of more potent azole drugs that might overcome CYP51-mediated resistance. Despite an incidence of azole resistance of about 3.5% for *C. albicans* clinical isolates and almost 30% for *C. glabrata*, thus far only *C. albicans* clinical isolates have been shown experimentally to confer azole resistance through mutations in CYP51. The reason for this difference is not known, but as *C. glabrata* is haploid, it ability to rapidly acquire mutated gain-of-function transcription factors that upregulate the expression of CYP51 and drug efflux pumps might provide a better alternative than target mutations that may result in less efficient CYP51s.

Of ~140 substitutions identified in CaCYP51 [95], most occur in combinations and only a few confer resistance. Other combinations give a functional enzyme in which azole resistance is enhanced additively or synergistically. Flowers et al. identified several single-site mutations in CaCYP51 that confer at least four-fold resistance to FLC. They showed that these mutations were located in proximity to the heme, the substrate entry channel, and the fungal specific loop (FSL) by using the crystal structure of full-length ScCYP51 in complex with lanosterol (PDB 4LXJ) [96]. We have used the CaCYP51-6×His structure to model seven single-site mutations located in the LBPs of azole resistant clinical isolates of *C. albicans* (Table 2). These mutations may directly or indirectly affect the binding of azole drugs. A second group not discussed here but described in Keniya et al. [124] is located outside the LBP and may affect indirectly the binding of azole drugs. Until crystal structures are obtained for these mutant CYP51s they are of less interest to drug discovery.

Mutations in the LBP may affect the binding of azole drugs directly. Consistent with its location in the mouth of the SEC, the A61V mutation affects binding of the long-tailed triazole PCZ but not FLC, VCZ, or ITC to LDM [124]. The CaCYP51 structure shows the alanine methyl group is about 4.3 Å from N3 of the 1,2,4-triazol-3-one group in the tail of ITC, while a valine methyl group might clash with the tail. It is likely that the hydrophobic valine methyl group would interact even more negatively with the bulkier and more polar tail of PCZ. This should not affect the binding of the shorter-tailed azoles FLC, VCZ and VT-1161 unless their access to the active site is impeded.

The ScCYP51-6×His Y140F/H active site mutations analogous to CaCYP51-6×His Y132F/H have been extensively characterized biochemically and structurally [120]. The CaCYP51 Y132F/H mutations result in FLC and VCZ resistant phenotypes [96,148]. Analysis using ScCYP51 shows this mutation will cause loss of a hydrogen bond between CaCYP51 Y132 and the heme ring C propionate as well as disrupting a water-mediated hydrogen bond network involving the tertiary alcohol of FLC, VCZ, and VT-1161 not found with ITC or PCZ.

Replication of the CaCYP51 Y132F/H mutations in ScCYP51 as Y140F/H gave the expected resistance phenotype in whole cell assays against FLC and VCZ and unchanged susceptibility for ITC and PCZ. Crystal structures of ScCYP51 in complex with FLC or VCZ identified a water-mediated hydrogen bond network between Y140 (equivalent to *C. albicans* Y132) and FLC or VCZ but not PCZ or ITC [120]. The Y140F mutation in ScCYP51 also displaced VCZ 0.5 Å closer to helix I within the active site. The structures of both full-length ScCYP51 and the CaCYP51 catalytic domain in complex with VT-1161 revealed a comparable water-mediated hydrogen bond network as VT-1161 has a tertiary alcohol group like FLC and VCZ [128]. Both enantiomers of the agrochemical prothioconazole-desthio have similar water-mediated interaction with ScCYP51 Y140 (PDB IDs: 5EAD, 5EAE) [106]. Mutations structurally aligned with Y140F/H also occur in CYP51s of the phytopathogens *Z. tritici*, *Mycosphaerella fijiensis* and *Uncinula necator* [150,151,152].

The conserved Y118 residue in the CaCYP51 active site is within 4 Å of ITC and forms a hydrogen bond with heme ring D propionate. The CaCYP51 Y118A mutation is expected to significantly increase the size of the active site adjacent to Y132, thereby reducing affinity for azole drugs, particularly the short-tailed azoles. These drugs bind entirely within the active site and are part of a water-mediated hydrogen bond network involving Y118, Y132, and both heme propionates.

The role of the conserved F126 residue in the CaCYP51 active site is poorly understood. Its phenyl group projects, parallel to helix I beside G303, to within 4 Å of ITC. The F126S mutation should increase the volume and polarity of the active site in proximity of the di-halogenated phenyl head group characteristic of most triazole drugs. This should reduce affinity for long- and short-tailed azole drugs, and most extensively for the latter. Conversely, the F380S mutation at the nexus of the SEC and PPEC should affect affinity for long-tailed azoles but possibly not for short-tailed azoles. The side chain of CaCYP51 G307S in helix I is predicted to clash with the di-halogenated head group of FLC or ITC and disrupt triazole binding to the heme [124]. This should modify the binding of similar triazole drugs.

The CaCYP51 I471 side chain is conserved in the *C. glabrata* and *S. cerevisiae* enzymes. It projects beyond the hydrophobic edge of the heme into the LBP surface bordered by helix C and helix I. The Y132H I471T mutations in the *C. albicans* Darlington strain confers azole resistance in a synergistic manner that is mimicked in *S. cerevisiae* LDM constructs. Modeling the ScCYP51 Y140H I471T structure suggests increased polarity in the active site beside the hydrophobic edge of the heme. How this confers synergistic resistance to the short-tailed but not long-tailed triazoles has yet to be elucidated. However, I471 is within 4 Å of K143, the lysine residue that becomes surface exposed on productive substrate binding. Could the I471T mutation enable the side chain of K143 to more efficiently bind its cognate NADPH-cytochrome P450 reductase, allowing the substrate lanosterol to compete more effectively with the short-tailed azole drugs in the active site?

AfCYP51A G54E/R/W mutations have been reported to confer resistance to the long-tailed triazoles PCZ and ITC [153,154]. Unexpectedly, MIC determinations which showed reproduction of these mutations in ScCYP51 gave phenotypes with wild-type susceptibility to FLC, VCZ and ITC. Crystal structures showing interactions between the mutated residues E73/W and the tail of ITC are likely to confer the susceptible phenotype [122]. ITC adopts different conformations within the LBP due to the flexibility of the piperazine ring in the tail of the inhibitor. The G73E/W mutations at the opening of the SEC result in ITC adopting a bent/twisted conformation (PDB IDs: 5ESG and 5ESH). PCZ has also adopts a bent shape in the *Trypanosoma brucei* CYP51 (PDB ID: 2 × 2 N and 2WV2) [155]. The twisted conformation of ITC in the ScCYP51 G73W mutant forced the F241 residue to move and close the PPEC off. Located at the nexus of the SEC and PPEC, ScCYP51 F241 is the only LBP residue conserved in ascomycete, basidiomycete and mucor-mycete but not human or plant CYP51s. This suggests it has an important but as yet unknown fungal specific function and that F241 might provide a useful contact point for the design of fungal-specific CYP51 inhibitors.

Comparison of crystal structures of AfCYP51B in complex with the inhibitor VNI (PDB ID: 4UYL) with ScCYP51 structures suggests the CYP51B SEC is of reduced size compared with the ScCYP51 and that the VNI overlays the PPEC of the *S. cerevisiae* enzyme. AfCYP51B G59 is equivalent to AfCYP51A G54. This residue is too distant to directly interact with VNI it as it does in ScCYP51. While mutations in G54 might confer resistance by altering entry to the active site, truncation of the transmembrane helix (TMH1) is likely to have significant effects on the conformation of residues around the SEC entry.

The crystal structure of the *C. albicans* LDM catalytic domain in complex with VT-1161 visualized a 2.5 Å hydrogen bond between the H377 (PDB ID: 5TZ1; equivalent to ScCYP51 H381) side chain and the oxygen in the tail of the drug [126]. Phylogenetic analysis, structural information and mutagenesis have been used to investigate the significance of this interaction. The structure of ScCYP51 in complex with VT-1161 (PDBID: 5UL0) showed the drug to be at a distance of 3.6 Å from H381, indicating a much weaker interaction [121]. Furthermore, the ScCYP51 H381A mutation conferred a weak increase in resistance to VT-1161. It has been claimed VT-1161 has good activity against a range of mucor-mycete pathogens [156,157]. but in these species the residue equivalent to ScCYP51 H381 or CaCYP51 H377 (Table 1) is replaced with a phenylalanine in both CYP51 F1 and F5 (Figure 5). In this case, π-stacking interactions between the benzene ring of this phenylalanine and the benzene ring in the tail of VT-1161 might be possible. A small hydrophilic pocket was identified in ScCYP51 at residues H381 and S382. The main chain amides of both residues and the carbonyl of S382 forming a hydrogen bond network with a cluster of three water molecules [120]. These residues are homologous to residues involved in forming a direct hydrogen bond and/or water-mediated hydrogen bond network with the 3-hydroxyl of lanosterol in complex with HsCYP51. One of the cluster waters forms a hydrogen bond with a nitrogen atom in the piperazine ring of the long-tailed triazoles ITC and PCZ (PDB IDs: 4ZDY and 4ZE1, respectively). Can this pocket be exploited to promote hydrophilic interactions with medium or long tailed azole drugs, or perhaps with transition state analogs of lanosterol?

In summary, crystal structures obtained with full-length ScCYP51, and the more recent structure for full-length CaCYP51, provide useful models to investigate resistance mutations in the LBP such as the CaCYP51 Y132F/H mutations. These crystal structures highlighted the conformational rigidity of the full-length structure in complex azole drugs and the roles of water molecules found in the active site and SEC. Because the binding of the substrate lanosterol can close off and slightly modify the active site of HsCYP51 [110], it is now important to carefully evaluate the conformational consequences of binding lanosterol and/or eburicol in the active site of full-length fungal CYP51. Such findings will be important for in silico ligand binding studies where ligand orientation in a predominantly hydrophobic environment is strongly affected by the neighboring water molecules capable of forming hydrogen bond networks. For example, by identifying hydrogen bond networks in the LBP, replacement of the difluoro-propanol linker of the tetrazole VT-1161 with the dioxolane linker from ITC overcomes the resistance to short-tailed azoles conferred by the Y140F/H mutations in ScCYP51. In addition, the importance of the transmembrane helix in CYP51 structures should not be overlooked. This is exemplified by the difference between the CaCYP51 catalytic domain structures in complex with VT-1161 and PCZ, specifically at the N-terminus (helix A′ and A) [121]. Since those helices contribute to the LBP, the truncation had its most significant effect when the medium-tailed VT-1161 was bound. Finally, the LBP of some CYP51s from other fungi may be too different in their composition to be represented ideally in homology models using ScCYP51 as template, e.g., AfCYP51A. This emphasizes the importance of obtaining full-length recombinant versions of such molecules for structural and functional analysis.

### 4.2. Screening Techniques for Antifungal Discovery

Difficult to treat bacterial diseases such as the tuberculosis and a range of parasitic diseases have provided useful models or drivers for the discovery of CYP51 inhibitors using either phenotypic or structure based approaches but with varying degrees of success. For example, Chagas disease, the most prevalent parasitic disease on the American continent, is caused by the protozoan *Trypanosoma cruzi*. Multiple generations of azole antifungals, including PCZ, have potent and selective in vitro activities against TzCYP51, but they were not curative in animal studies. Lepesheva’s group used a high throughput microplate-based spectroscopic screen of Type II binding to identify imidazoles (including VNI and VNF) and an aniline (Chemdiv C155-0123) with strong heme-dependent affinity for TzCYP51 [4,158]. Additional biochemical assays were then used to show VNI and VNF were functionally irreversible ligands not outcompeted by the substrate molecules of this target and that they were not effective against HsCYP51. Chemdiv C155-0123, also identified independently in a screen of *Mycobacterium tuberculosis* CYP51 [159], was found to selectively bind TzCYP51 and provide partial cures of acute Chagas disease. VNI and VNF substantially overlap PCZ in their positioning within the active site and SEC, while a derivative of C155-0123 has its biaryl tail instead occupying a hydrophobic tunnel adjacent to the F-G loop and a two stranded β-sheet near the C-terminus (comparable to the PPEC in *S. cerevisiae*). The indole ring of the C155-0123 biaryl derivative locates within the hydrophobic region occupied by the difluorophenyl group of PCZ adjacent to helix I and could be extended with derivatives that enter the space occupied by the dichlorophenyl-oxyphenyl group of difenoconazole and the chloro-diphenyl group of VNF.

Several studies have identified antifungal compounds and then used in silico docking to suggest how they might interact with CYP51. In some cases, the research has been extended using molecular dynamics simulations. For example, Lebouvier et al. [160] identified *R* and *S* enantiomers of 2-(2,4-dichloropenyl)-3-(1*H*-indol-1-yl)-propan-2-ol as antifungal and found the 100-fold more active *S* enantiomer gave MIC values from 0.2–167 ngm/mL for a range of *Candida* species. While docking studies and molecular dynamics simulations were used to justify the preferential binding of the *S* enantiomer, a failure to consider the likely presence of a water-mediated hydrogen bond network between CaCyp51 Y132 and the tertiary hydroxyl in the ligand, as shown with the crystals structures of CaCYP51 and ScCYP51 in complex with VT-1161 or ScCYP51 in complex with FLC and VCZ, was an important deficiency. Zhao et al. used molecular docking of two antifungal isoxazole compounds with AfCYP51B to suggest that their activity was dependent on hydrogen bond interactions between the isoxazole ring oxygen and Y122 [161]. They then focused on identifying biphenyl imidazoles with antifungal activity and used molecular modelling to suggest, despite their lack of activity against *A. fumigatus*, that the 2-fluorine of the biphenyl would form a hydrogen bond with the Y122 of CYP51B [162]. The same residue is conserved among fungal pathogens and is equivalent to the Y126 in ScCYP51 and Y118 in CaCYP51. Binjubair et al. [163] assessed the activity of a range of short and extended derivatives of *N*-benzyl-3-(1*H*-azol-1yl)-2-phenylpropionamide against the sequenced strain of *C. albicans* (Sc5314) and the clinical isolate (CaI4). They also measured the type II binding to CaCYP51 and HsCYP51 and inhibition of these enzymes in vitro with the most active of these compounds in order to demonstrate selectivity. Docking and molecular dynamics studies showed that the short derivatives bound in the active site of the CaCYP51 (PDB 5FSA) in a manner comparable to FLC while an extended derivative bound similarly to PCZ but with its tail in the SEC, making unique interactions via its benzene ring and a sulphonamide group. Rabelo et al. [10] have summarized several studies of compounds with a head grouping comprising a tertiary alcohol linking triazole plus a difluoro-phenyl group. Multiple derivatives showing strong antifungal activity against a range of fungal pathogens were docked with various models of CYP51. These studies usually located the head group in the active site, with the triazole interacting with the heme and the derivative tail within the SEC. A study by de Almeida et al. [164] tested the activity of diphenyl-phosphane derivatives of ketoconazole and used poses found with a model of truncated CaCYP51 in complex with PCZ to ask how the derivatives bound to the enzyme. Although the docking study correctly modeled the most features affecting the binding of PCZ, the conformation of the triazole group interaction with the heme did not match with crystal structures. Such a fundamental difference calls into question conclusions based on the docking analysis. Crystallization of such triazoles with ScCYP51 could be informative and assist the design of the more effective SEC interacting tails. Clearly, comprehensive biochemical analysis of ligand efficacy and the use of liganded crystal structures obtained with a robust system such as full-length ScCYP51 would provide significantly more reliable information than in silico analysis.

### 4.3. Use of In Silico Methods to Identify Ligands of CYP51

The available high-resolution crystal structures of fungal CYP51s make possible the use of in silico methods to screen compound libraries, libraries of drug-like fragments and even theoretical ligand structures for potential interactions as substrates or inhibitors with the enzyme by using software suites such as Schrödinger Prime. By using either structure-based docking or pharmacophore-based selection, millions of compounds can be screened, and potential ligands ranked in order to identify compounds worth purchasing or synthesizing for in vitro and in vivo tests of efficacy. This cost-saving approach will be enhanced in value by growing the library of crystal structures of CYP51s freely available from the PDB for major fungal pathogens of humans and fungal phytopathogens and with a variety of ligands. Importantly, the liganded structures also provide key tests of software and assumptions used, i.e., do docked ligands accurately reproduce binding modes found in crystal structures of CYP51-ligand complexes?

Fragment-base discovery applied to *M. tuberculosis* CYP121, followed by fragment merging, overcoming the internal strain generated by fragment merging, plus further synthetic merging and optimization, identified an inhibitor with good active site occupancy and a *K*_D_ = 15 nM [165,166,167]. While this study illustrates the potential of fragment-based discovery when integrated with the ability to visualize binding and measurements of candidate inhibitor activity, its affinity for its target and its activity against drug metabolizing liver enzymes, none of the lead compounds showed growth inhibitory activity when tested in vitro against an Mtb strain. Whether further modification of these candidates will allow effective inhibition, perhaps by overcoming problems with cell permeability or drug efflux pumps, awaits further research.

Kritsi et al. [168] used a pharmacophore screen based on five imidazole and triazole drugs to identify 1 million compounds meeting drug-likeness criteria within the 11 million compound ZINC database. They then used in silico docking with a homology model of AfCYP51A to identify eight non-azole compounds, including four that appeared to dock in close proximity to the heme iron and aromatic amino acids in the active site. However, *A. fumigatus* ATCC 204303 and a clinical isolate showed MIC values for all compounds that were greater than 100 µM. While this is an encouraging advance, the compounds identified will require considerable optimization to be of practical value.

A successful example of an in silico approach to antifungal discovery used a homology model of AfCYP51A liganded with PCZ (developed from the crystal structure of TzCYP51 in complex with the same ligand) to identify analogs of VT-1161 with more extensive interactions with the SEC. This led to VT-1598, which has broader spectrum antifungal activity than VT-1161, including strong activity against yeast, dermatophytes and molds like *A. fumigatus* [169]. A similar approach also helped realize the value of extending the tail of the trypanosomal CYP51 inhibitor VNI with a trifluoro-ethoxy substituent [158]. This VNI derivative has increased potency and a broader spectrum of antifungal activity. It is highly active in vitro against *C. albicans* and *A. fumigatus* and has been shown to bind tightly within the LBP.

In silico screens rely on the quality of the structural models used and the constraints invoked. Although homology modeling can be used to predict the 3D structure of proteins, it is critical to select a closely related template and achieve robust alignment of sequences. The availability of crystal structures for full-length *S. cerevisiae*, *C. glabrata* and *C. albicans* CYP51s, as well as crystal structures for the catalytic domain of CaCYP51 and AfCYP51B provide much more reliable templates than earlier efforts based on the structures of CYP51s from the catalytic domain of the human enzyme, or the *M. tuberculosis* CYP51. However, comparison of truncated and full-length LDM crystal structures shows, for truncated versions of the enzyme, that the conformation of the mouth of the SEC is affected by the length of the channel occupied by the ligand [121]. Molecular docking experiments that do not include key water molecules in the active site may affect the orientation of triazoles in this region despite binding to the heme iron. For example Shi et al. [170] produced binding profiles that fail to take into account the water-mediated hydrogen bond networks that interact with the tertiary alcohols of FLC and VCZ and with a piperazine ring nitrogen of ITC and VCZ. While they concluded that the long-tailed azoles showed higher affinity binding due to larger numbers of interactions with the LBP, and particularly the SEC, they also reached the surprising conclusion that polar interactions are relatively unimportant for the binding of FLC and VCZ. A recent study by Sari and Kart [171] is instructive because it compared four Glide molecular docking protocols with the crystal structure obtained for CaCYP51 in complex with VT-1161. This was done by redocking VT-1161 in the active site of the CaCYP51 (PDB 5TZ1) model and calculating the RSMD values for the best pose with respect to the co-crystallized binding conformation of VT-1161. The RMSD values obtained were in the range of 0.51 to 0.77 Å, with the QM-Polarised Ligand Docking method giving the lowest RMSD value and the closest representation to the crystal structure, in part due to improved interactions with the heme iron. Finally, as there is significant variation between fungal pathogens in the amino acids contributing to the LBP, the value of in silico screening methods could be strengthened significantly by obtaining further high resolution crystal structures of fungal and host CYP51s. Some key examples include full-length structures of AfCYP51A and CYP51B, *C. neoformans* CYP51, representatives of the mucor-mycete CYP51s such as *R. arrhizus* F1 and F5, and human cytochrome P450s including HsCYP51and liver cytochrome 450s such as CYP3A4.

Better understanding of the CYP51 catalytic mechanism suggests additional sites to which inhibitors could be targeted. These include the PPEC where CYP51 appears to provide a template for its product to undergo subsequent steps in the ergosterol biosynthetic pathway mediated by the Ergosome [118,130]. A possible strategy would be the design of suicide mimics of the LDM product. The NADPH-cytochrome P450 reductase binding site near the fungal specific loop, the heme bulge and helix C may provide a drug target. This could be assayed by using carbon monoxide binding to detect inhibition of heme reduction by the cognate NADPH-cytochrome P450 reductase or by using GC-MS methods to assay the LDM reaction. A simpler alternative may be to use the BOMCC assay of Riley et al. [172], not only for this purpose but also to assess either substrate binding or the inhibition of membrane bound fungal CYP51s and baculosome preparations of the liver enzyme such as cytochrome 3A4. With BOMCC having a Km of ~70 µM at pH 8, assay of membrane preparations containing <100 nM concentrations of CYP51 enzymes can provide more reliable estimates of drug affinities in the nanomolar range than is possible using type II binding experiments. Type II binding experiments are problematic because they require application of the Hill equation or the rearranged Morrison equation to essentially 1-1 binding interactions that use ≥1 µM enzyme.

Inhibitors need to be of sufficient affinity to block substrate binding. For example, FLC has affinities with its target CYP51s in the low µM range and is therefore competitive with substrates such as lanosterol and eburicol. The triazoles VCZ, ITC and PCZ and the tetrazoles VT-1161, VT1129 and VT-1598 each bind within the CYP51 active site but are all effectively non-competitive inhibitors because their affinities are in the nanomolar range. The greater lipid solubility of some azole drugs, such as ITC and PCZ, is likely to make them are more effective because are they concentrated in membranes.

As an alternative to type II binding and enzyme activity measurements, surface plasmon resonance can be used determine the kinetic constants for the association (*k_on_*) and dissociation (*k_off_*) of inhibitors and substrates for affinity purified LDM covalently bound to an optical biosensor. Because the dissociation constant (*K_d_*) is equivalent to a ratio of these two kinetic measures (*K_d_* = *k_off_*/*k_on_*), it can be measured independent of the amount of enzyme bound to the detector surface. Binding recombinant CYP51s to the sensor surface enables effective measurement of the sub-µM *K_d_* values for larger azole drugs such as PCZ but is of insufficient sensitivity to detect the binding of some smaller azole drugs such as FLC.

The future use of azole antifungals is likely to be limited by their intrinsic ability to elicit antifungal resistance primarily due to multiple exposures during prophylaxis, therapy and crop protection that result in the development of target site mutations, CYP51 overexpression, and the induction of drug efflux pumps. In principle, some of these problems could be overcome by:

1. Using smart design to obtain potent azoles with low nanomolar affinities that prevent competition with substrate(s) in the LBP,
2. Converting fungistatic azole drugs into rapid acting fungicides by using combinations with inhibitors of ABC and MFS drug efflux pumps such as clorgyline [173],
3. Discovering azoles that are not substrates of drug efflux pumps and/or do not affect transcriptional regulators responsible for upregulation of *ERG11* or drug efflux pumps [50], and
4. Designing bifunctional azoles that inhibit targets in ergosterol biosynthesis in addition to CYP51—such as squalene epoxidase [174] or C24-methyl transferases [175].

Interestingly, the azole resistance associated with the rare inactivation of *C. albicans* Erg3 might be overcome by using Lovastatin in combination with ITC [176]. This appears to occur via inhibition of HMG-COA reductase and increased *ERG3* expression.

## 5. Future Directions

Structural studies have provided a wealth of knowledge about fungal and host CYP51 enzymes in the form of high-resolution crystal structures with many ligands of interest, plus model structures using these crystal structures as templates. These structures, together with knowledge of mutations that confer azole resistance, provide tools that allow the design of improved fungal CYP51 inhibitors and the in silico exploration of chemical space in order to identify candidates for testing as antifungals. Subsequent research focusing on such compounds needs access to a variety of phenotypic screens, biochemical assays and ancillary techniques in order to test their efficacy. From our practical experience, this process is likely to require in vitro assays that include type I substrate binding and type II drug binding using affinity purified recombinant CYP51s, as well as substrate-based or BOMCC-based assays that use crude membranes from yeast strains that co-express CYP51s of interest together with their cognate NADPH-cytochrome P450 reductase. Commercially available baculosome preparations co-expressing liver cytochrome P450 such as CYP3A4 and a suitable cognate NADPH cytochrome P450 reductase enable parallel in vitro tests of compound selectivity. These biochemical approaches can, for example, be complemented with phenotypic tests that use agarose diffusion drug susceptibility assays and MIC determinations with *S. cerevisiae* strains overexpressing CYP51 targets from a range of fungi, drug efflux pumps or transcriptional regulators, plus MIC determinations obtained with well-characterized clinical isolates. Cycles of refinement should also include, where possible, crystal structures of drug candidates in complex with their CYP target or with a robust surrogate such as *S. cerevisiae* CYP51. As suggested by Rabello et al. [10], the early evaluation of safety profiles using industry standard in silico ADMETox tests of antifungal candidates is an invaluable way to eliminate problematic drug candidates and make optimization strategies more efficient. The best broad spectrum antifungal candidates, with suitable computed ADMETox properties, can then be taken forward in tests of toxicity using models such as nematodes or moth larvae, and then with culture cells, before considering implementation of trials in animal or plant models.

## Figures and Tables

**Table 1:** Amino acid residues contributing the ligand-binding pocket in crystal structures of full-length fungal lanosterol 14α-demethylases (LDMs).

SRS Number	*S. cerevisiae* CYP51 + ITC	*C. glabrata* CYP51 + ITC	*C. albicans* CYP51 + ITC
1 (SEC&PPEC)	*A*124*A**Y***A*HLT*TPV***F***GKGV***IY***DCP143	*A*125*A**Y***S*HLT*TPV***F***GKGV***IY***DCP144	*D*116*A**Y**KHLT*TPV***F***GKGV***IY***DCP135
4 (Helix I)	I304AN*L*LI***GV***L*MG*GQ*H**T***SAA321	I305AN*L*LI***GV***L*MG*GQ*H**T***SAA322	I297AN*L*LI***GI***L*MGG*Q*H**T***SAS314
5 (Interior loop)	H378*P**L****HS***(*L*)***F***R385	H379*P**L****HS***(*L*)***F***R386	M374*P**L****HS***(*I*)***F***R381
6 (SEC)	D505(***FT***)**S*****M**V*(*T*)LPTG515	D508(***FT***)***SM**V*(*T*)LPTA518	D504(***YS***)***SM**V*(*V*)LPTE514
Outside SRSs but <4 Å of ITC	***A***69(***V***70) ***Y***72***G***(*M*74), ***F***236, ***P***238, ***F***241	***A***70(*I*71) ***Y***73***G***(*T*75), ***F***237, *P*239, ***F***242	***A***61(*A*62) *Y*64***G***(*Q*66), ***F***228, ***P***230, ***F***233
Additional residues in internal surface of active site, SEC & PPEC	*L*95*L*96, *R*98, *M*100, *L*147, *Q*150*K*151, *V*154, *I*239, *V*242, *H*405, *I*471	*L*96*L*97, *R*99, *M*101, *L*148, *Q*151*K*152, *V*155, *I*240, *V*243, *H*406, *I*473	*P*68 *L*87*L*88, *K*90, *M*92, *L*139, *Q*142*K*143, *A*146, *A*149*L*150, *Y*158, *L*204, *L*276, *I*231, *V*234, *Y*401, *I*471

SRS1 and SRS4-6 are “substrate recognition sites” as defined by Warrilow (reviewed in [7]. Residues in italics contribute to the interior surface of the LBP i.e., the active site, the SEC and the PPEC (8). Residues within 4 Å of ITC are in boldface. Y64 in CaCYP51 is within 4.1 Å of ITC. Non-identical structurally aligned residues contributing to the LBP within 4 Å of ITC are shown in brackets. LBP residues differing either chemically or in conformation compared with the reference structurally aligned ScCYP51 residues are highlighted in yellow. Forty-eight residues contribute the interior surface of the LBP of CgCYP51 and ScCYP51. A further 5 CaCYP51 residues (A149, L150, Y158, L204 and L276) may contribute to a minor extension artifact in the LBP while K118 at the external edge of the PPEC and P68 beside the water-containing pocket in the SEC may also contribute very small areas to the LBP surface. Single mutations at 8 LBP residues (highlighted in purple) have been shown to contribute to azole resistance in *C. albicans*. No equivalent mutations have been reported for CgCYP51. The six residues underlined are found in major ascomycete and basidiomycete fungal pathogens but not in their human or plant hosts. These residues have yet to be shown to confer resistance to azole drugs. Of these residues, only the residue aligned with ScCYP51 F241 is conserved in the mucormycetes. LBP, ligand-binding pocket. SEC, substrate entry channel. PPEC, putative product exit channel.

**Table 2:** Single amino acid substitutions in *C. albicans* CYP51 LBP that confer azole resistance.

Mutation	Predicted Effect
*A61V*	Modified mouth of substrate entry channel (SEC) affects the binding of long-tailed azoles
***Y118A***	Enlargement of LBP beside heme ring D propionate confers loss of water-mediated H-bond interactions with tertiary alcohol of FLC, voriconazole (VCZ), and VT-1161 and heme ring D propionate
*F126S*	Enlarged and more polar LBP in helix B beside helix I G303
***Y132F/H*** ^a,b,c^	Confers loss of both H-bond with heme ring C propionate and water-mediated H-bonds with tertiary alcohol of FLC, VCZ and VT-1161
**K143R/Q**	Modification of side chain involved in ionic bond with heme ring C propionate and conformation of heme bulge affected
***G307S***	Formation of helix I S307-OH H-bond to triazole group affected
*F380S*	Enlargement and increased polarity of the nexus of SEC and putative produce exit channel (PPEC)
**R467K** ^a,c^	Possible K467 side chain interaction with N136 may affect main chain H-bond with K143 side chain
**I471T** ^a,b^	Increased polarity in environment beside K143, helix I and the heme ring C propionate.

Single mutations found in the LBP of CaCYP51 azole-resistant clinical isolates are shown. Mutated residues within 4 Å of ITC are in italics. Mutations shown to confer azole resistance by expression in *S. cerevisiae* are shown in bold. Mutations that were considered included references [95,96,116,144,145,146,147,148,149]. ^a^ Mutations shown to affect azole binding, substrate affinity, and/or enzyme activity. ^b^ Mutations investigated using structurally aligned residues in ScCYP51-6×His. ^c^ Mutations investigated by expressing mutant CYP51 in a drug-susceptible *C. albicans* host.

## Data Availability

No new data were created or analyzed in this study. Data sharing is not applicable to this article.
