# Correction: Monk, B.C.; Keniya, M.V. Roles for Structural Biology in the Discovery of Drugs and Agrochemicals Targeting Sterol 14α-Demethylases. *J. Fungi* 2021, *7*, 67

**DOI:** 10.3390/jof7121011

**Published:** 2021-11-26

**Authors:** Brian C. Monk, Mikhail V. Keniya

**Affiliations:** Department of Oral Sciences, Sir John Walsh Research Institute, University of Otago, Dunedin 9016, New Zealand; mikhail.keniya@otago.ac.nz

In the original publication, there was a mistake in Figure 4. *Lanosterol in the ligand-binding pocket (LBP) of CYP51* that was published [1]. The arrows retained a white instead of a transparent background and thus obscured parts of the figure. The corrected Figure 4 appears below. The authors apologize for any inconvenience caused and state that the scientific conclusions are unaffected. The original publication has also been updated.

## Figures and Tables

**Figure 4:**
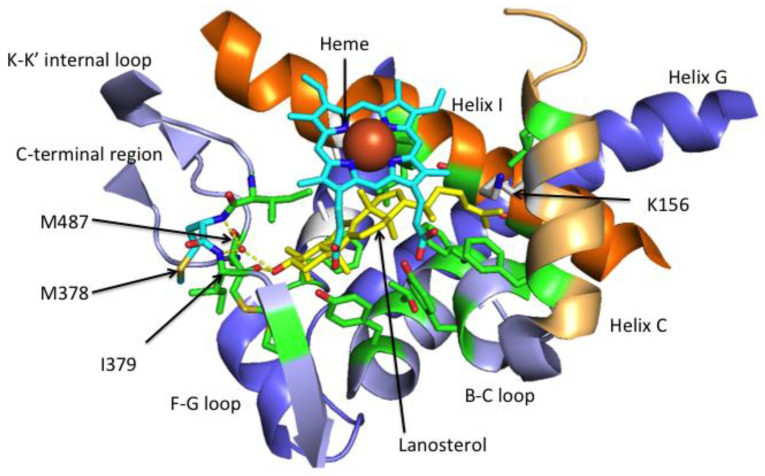
Lanosterol in the ligand-binding pocket (LBP) of CYP51. Relevant portions of the human HsCYP51 crystal structure (Protein Data Bank (PDB) 6UEZ) are shown. Residues within 4 Å of lanosterol (yellow) are shown with carbon atoms in green. These 16 residues are found in helix I, helix B, helix C, the B-C loop, the K-K’ internal loop and the C-terminal region. Proton channel S mutations, D231A in helix F and H314A in nearby helix I, are shown in white. Upon lanosterol binding helix C has changed conformation slightly, the K156 side chain loses its ionic interaction with the heme propionate C and becomes exposed into the enzyme surface. The K-K’ internal loop I379 main chain carbonyl hydrogen bonds with the OH of lanosterol. The main chain amides of M378 and I379 plus the main chain carbonyl of M487 in the C-terminal region form a water-mediated hydrogen bond network with the hydroxyl of lanosterol. The 14α-methyl group of lanosterol lies in proximity of the heme iron (large red ball) in a catalytically competent position.
